# Novel Adsorption–Reaction Process for Biomethane Purification/Production and Renewable Energy Storage

**DOI:** 10.1021/acssuschemeng.1c06844

**Published:** 2022-06-07

**Authors:** Joana A. Martins, Carlos V. Miguel, Alírio E. Rodrigues, Luis M. Madeira

**Affiliations:** † LEPABE, Laboratory for Process Engineering, Environment, Biotechnology and Energy, Chemical Engineering Department, Faculty of Engineering, University of Porto, Rua Dr. Roberto Frias, 4200-465 Porto, Portugal; ‡ LSRE - LCM, Laboratory of Separation and Reaction Engineering - Laboratory of Catalysis and Materials, Chemical Engineering Department, Faculty of Engineering, University of Porto, Rua Dr. Roberto Frias s/n, 4200-465 Porto, Portugal; § ALiCE, Associate Laboratory in Chemical Engineering, Faculty of Engineering, University of Porto, Rua Dr. Roberto Frias, 4200-465 Porto, Portugal

**Keywords:** Biogas upgrading, Decarbonization, Multifunctional reactors, Renewable methane, Carbon capture and utilization, CO_2_ methanation, Substitute natural gas, Power-to-gas

## Abstract

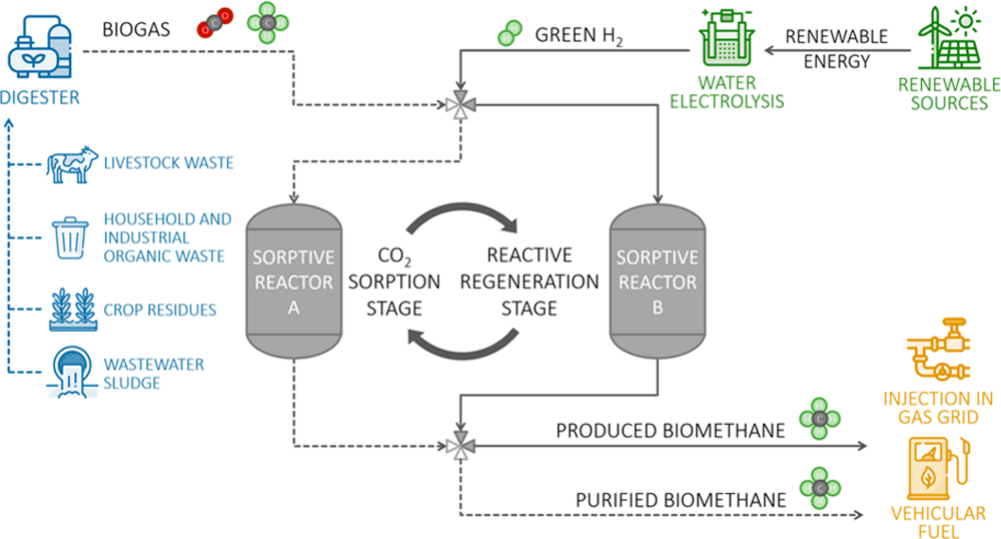

This work proposes an innovative method for the simultaneous upgrading of biogas streams and valorization of the separated CO_2_, through its conversion to renewable methane. To this end, two sorptive reactors were filled with a layered bed containing a CO_2_ sorbent (K-promoted hydrotalcite) and a methanation catalyst (Ru/Al_2_O_3_). The continuous cyclic operation of the parallel sorptive reactors was carried out by alternately feeding a biogas stream (CO_2_/CH_4_ mixture) or H_2_. The CO_2_/CH_4_ mixture is fed to the sorptive reactor during the sorption stage, with CO_2_ being captured by the sorbent and CH_4_ exiting as a purified stream (i.e., as biomethane). During the reactive regeneration stage, the inlet stream is switched to pure H_2_, which reacts with the previously captured CO_2_ at the methanation catalyst active sites thus producing additional methane. For continuous operation, the two sorptive reactors were operated 180° out of phase and cyclic steady-state could be reached after ca. five cycles. The performance of the cyclic sorptive-reactive unit was assessed through a parametric study to evaluate the influence of different operating conditions, namely, the inlet flow rate and CO_2_ content during the sorption stage, the hydrogen inlet flow rate during the reactive regeneration stage, the stage duration, and temperature. The inclusion of an inert purge after the reactive regeneration stage was also tested. The performance of the unit was compared to the case of direct hydrogenation of biogas, and conclusions were drawn regarding future optimization, with special attention being given to CH_4_ productivity and purity. During the parametric study, a compromise between these process indicators, i.e., a productivity of 1.63 mol_CH4_ kg_cat_^–1^ h^–1^ with 70.3% of CH_4_ purity, was obtained at 350 °C. However, biomethane purities above 80% were easily achieved, though at the expense of methane productivities.

## Abstract 2

The method proposed contributes to sustainability by aggregating the upgrading of biogas with the valorization of separated CO_2_, through its conversion to renewable methane.

## Introduction

In its latest report, the International Energy Agency disclosed that the annual worldwide production of biogas is 35 MToe (million tons of oil equivalent).^1^ This value is expected to reach 92 MToe by 2030 and 151 MToe by 2040; however this is still very far from what is reported to be the full sustainable potential of biomethane (730 MToe), which could cover ca. 20% of today’s worldwide gas demand.^1,2^

Biogas is the product of the anaerobic digestion of organic compounds and so, in addition to being a well-established process for the generation of renewable energy, it is also a route for the treatment of organic wastes, contributing to a circular economy.^3^ Biogas can be generated in sewage treatment plants, landfills, or other sites for industrial and agricultural waste processing. The biogas composition depends mainly on the nature of the substrate and the operating conditions of the digestion.^4,5^ Its primary components are CH_4_ (typically 40–75%) and CO_2_ (ca. 15–60%). Other minor components such as N_2_, H_2_O, O_2_, H_2_S, NH_3_, CO, siloxanes, and halogenated hydrocarbons are usually also present and can be removed through various cleaning processes.^5,6^ Biogas upgrading consists of the separation of CO_2_ and aims to increase the low calorific value of the biogas, converting it to a higher standard fuel, biomethane.^3^ The state of the art technology for biogas upgrading includes scrubbing processes based on amines, organic solvents or water, cryogenic distillation, membrane separation, pressure-swing adsorption, and biological methane enrichment.^3−6^ After its upgrading to biomethane, biogas finds more applications, for instance, in households (through injection in the gas grid) as well as a vehicular fuel.^4^ Most implemented techniques for biogas upgrading are merely separation processes, thus originating a CH_4_-rich stream (biomethane), and a CO_2_-rich stream.

The CO_2_-rich stream that arises from biogas upgrading can be converted into more methane, for instance, in the scope of power-to-gas processes.^7^ The power-to-gas concept envisions the production of green H_2_ from renewable power via water electrolysis. The green H_2_ may then be further converted into methane through the Sabatier (or methanation) reaction, presented in eq 1.^7−9^ In comparison with other fuels, the substitute natural gas (CH_4_) is a high-energy-density gas that benefits from a well-established distribution and storage infrastructure, the natural gas grid, making it a relevant product and thus attracting interest.^7,8,10^ If such an approach is considered, i.e., if the CO_2_ removed during biogas upgrading and renewable H_2_ are catalytically converted, the amount of methane generated from an initial biogas stream (composed, for instance, of 50% CH_4_ and 50% CO_2_) can be virtually doubled.
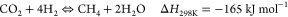
1

A simple process of direct hydrogenation of CO_2_ (simultaneous feeding of biogas and renewable hydrogen to the methanation reactor) may be considered, but the reversible nature of the Sabatier reaction (cf. eq 1) implies that the presence of CH_4_ in the feed is unfavorable, often requiring multiple reactors to achieve the desired CO_2_ conversions and CH_4_ purity.^11,12^ One of the alternatives reported in the literature envisions the use of hybrid reactors (sorption-enhanced or membrane reactors) that, through the removal of a methanation product, namely H_2_O (using a selective sorbent or membrane), shifts the reaction equilibrium toward the formation of more CH_4_. These are interesting approaches but may entail heat management issues (given the highly exothermic nature of the methanation reaction) and present a low TRL (technology readiness level), still requiring further improvements, mainly with regard to the stability and selectivity of membranes at temperatures ideal for the methanation reaction.^13−17^

The enhanced upgrading strategy presented in this work conceives the use of a single unit, wherein CO_2_ capture and its conversion into CH_4_ are carried out in the same device. For this end, the reactor filling (to which the biogas and H_2_ streams are fed) must consist of a mixed bed containing CO_2_ sorbent and methanation catalyst or a dual-function material with both capabilities.^18−24^ The most common methanation catalysts consist of an active metal, typically Ni, Ru, or Rh dispersed on a metal oxide support such as Al_2_O_3_, SiO_2_, or TiO_2_. Ruthenium-based catalysts have been reported to be active and CH_4_-selective even at low temperatures.^9,25,26^ Also, according to the literature, Ru catalysts are more resistant to deactivation at low temperatures in comparison to Ni, as Ni metal particles interact with CO, forming mobile nickel sub-carbonyls that may lead to the loss or sintering of the Ni particles.^27,28^ With regard to CO_2_ sorbent materials, the most common are zeolites, activated carbons, calcium oxides, hydrotalcites, organic–inorganic hybrids, and metal–organic frameworks.^29−31^ Hydrotalcites are particularly relevant for the described application, since they are compatible with the methanation catalysts in terms of the temperature of operation (200–400 °C) and have high selectivity for CO_2_ over gases such as CH_4_, CO, and N_2_, but also present fast sorption kinetics, are able to undergo easy regeneration, and are stable over cycles with a low loss of sorption capacity.^32,33^

The CO_2_ capture and conversion to CH_4_ in sorptive reactors has been tested and reported before, but only for the removal of CO_2_ from simulated flue gas streams (composed essentially of CO_2_ and N_2_, but also of H_2_O and O_2_), and never for CO_2_/CH_4_ mixtures (e.g., biogas streams). Particularly, Miguel et al. used one single sorptive reactor for CO_2_ capture from flue gas (whose composition was simplified to N_2_ and CO_2_) and its catalytic conversion to CH_4_ via the Sabatier reaction, in the same device. In their work, the concept was tested at the laboratory scale, under different pressure and temperature conditions. The sorptive reactor, filled with a mixture of CO_2_ sorbent and methanation catalyst, was alternately fed with a simulated flue gas stream (15% CO_2_ in N_2_), during the sorption stage, and an H_2_ stream during the reactive regeneration stage.^34^

**Figure 1:**
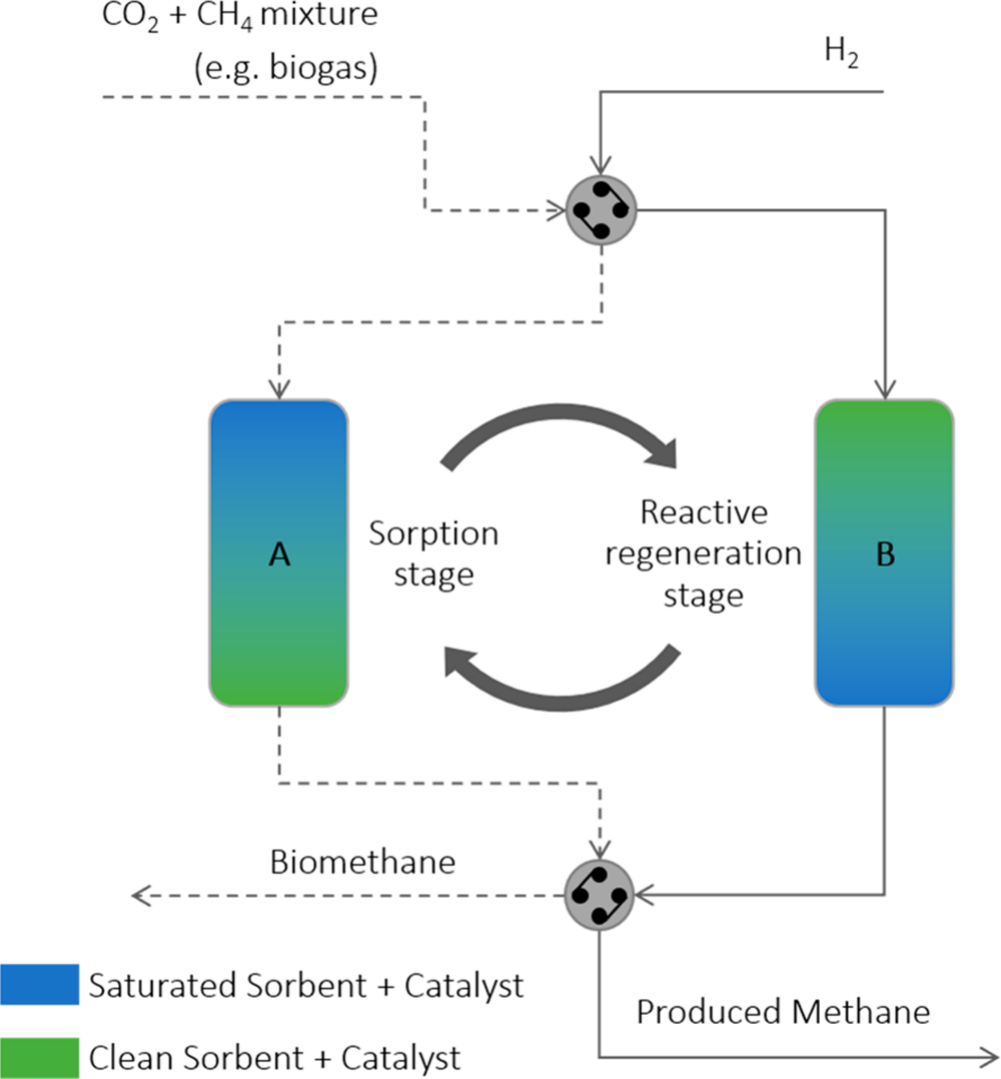
Schematic illustration of the proposed process for biogas upgrading, using two sorptive reactors (A and B). In the scheme, sorptive reactor A is in the sorption stage (its inlet is composed of biogas) and sorptive reactor B is in the reactive regeneration stage (inlet composed of pure H_2_).

However, for the process to run continuously, at least two reactors are required, as presented in Figure 1. In this scheme, one of the sorptive reactors (A) is in the sorption stage and so, as it is being fed with the CO_2_/CH_4_ mixture, the CO_2_ is captured and CH_4_ exits as (purified) biomethane. Simultaneously the remaining sorptive reactor (B) is in the reactive regeneration stage and therefore it is fed with pure H_2_ which, upon reaction with the previously captured CO_2_, is converted to more methane. As the CO_2_ sorbent becomes increasingly saturated in reactor A, the inlet streams are switched and the stages of the sorptive reactors are inverted; sorptive reactor A initiates the reactive regeneration stage and sorptive reactor B starts the sorption stage. The cycling of the system is perpetuated by the actuation of upstream and downstream valves, thus creating the described sorption/reactive regeneration cycles.^35^

During the operation of the described sorptive reactors, the exothermic methanation reaction (cf. eq 1) occurs simultaneously with the endothermic desorption of CO_2_. Hence, the utilization of such a concept allows mitigating the risks of poor heat dissipation and enhancing heat management and temperature control, which are key parameters in the design and operation of methanation reactors. The proposed cyclic unit also confers versatility to the operation because, instead of only one outlet stream, it generates two (“Biomethane” and “Produced Methane” in Figure 1), which may have different compositions. The two streams can be used separately, for instance, the purest stream may be injected into the natural gas grid and the second can be stored and/or used for self-consumption (e.g., combined heat and power generation), or they can be mixed.

In this work, a cyclic unit composed of two parallel sorptive reactors filled with a mixture of a commercial CO_2_ sorbent (K-promoted hydrotalcite) and a commercial methanation catalyst (Ru/Al_2_O_3_) was tested. To the authors’ knowledge, no similar cyclic unit has been studied before for the continuous and simultaneous biogas upgrading and valorization of the separated CO_2_ to CH_4_. Herein, and according to the method previously described, the sorption and reactive regeneration stages were performed alternately on both sorptive reactors until a cyclic steady-state was achieved. In order to assess the effect of operating conditions on the capture and conversion of CO_2_, a parametric study was performed in which several values of inlet flow rate and CO_2_ content during the sorption stage, inlet flow rate during the reactive regeneration stage, stage duration, and temperature were tested. Additionally, the inclusion of an inert purge step after the reactive regeneration was also considered. Each experiment was evaluated by several process indicators, namely, CO_2_ sorption capacity, CO_2_ conversion, CH_4_ productivity and purity, and moles of H_2_ fed per mole of CH_4_ produced. Finally, the proposed method was compared to the direct hydrogenation of biogas and conclusions were drawn with regard to the path for the future optimization of the unit.

## Experimental Section

### Parametric Study

#### Experimental Setup

In this work two stainless-steel reactors with a length of 18 cm and an internal diameter of 5.9 cm were packed with (i) a commercial CO_2_ sorbent, a K-promoted hydrotalcite from SASOL (PURAL MG30K)—presented as cylindrical pellets with a length and diameter of 4.7 mm, (ii) a methanation catalyst, 0.5% ruthenium on alumina from Sigma-Aldrich—presented in cylindrical pellets with a length and diameter of 3.2 mm, and (iii) inert glass spheres with a 5 mm diameter, also from Sigma-Aldrich. The materials were placed inside the reactor in alternate layers of catalyst and CO_2_ sorbent. All layers were diluted in inert glass spheres, and the overall catalyst to sorbent ratio was 1:5. The bottom layer was a catalyst layer.

**Figure 2:**
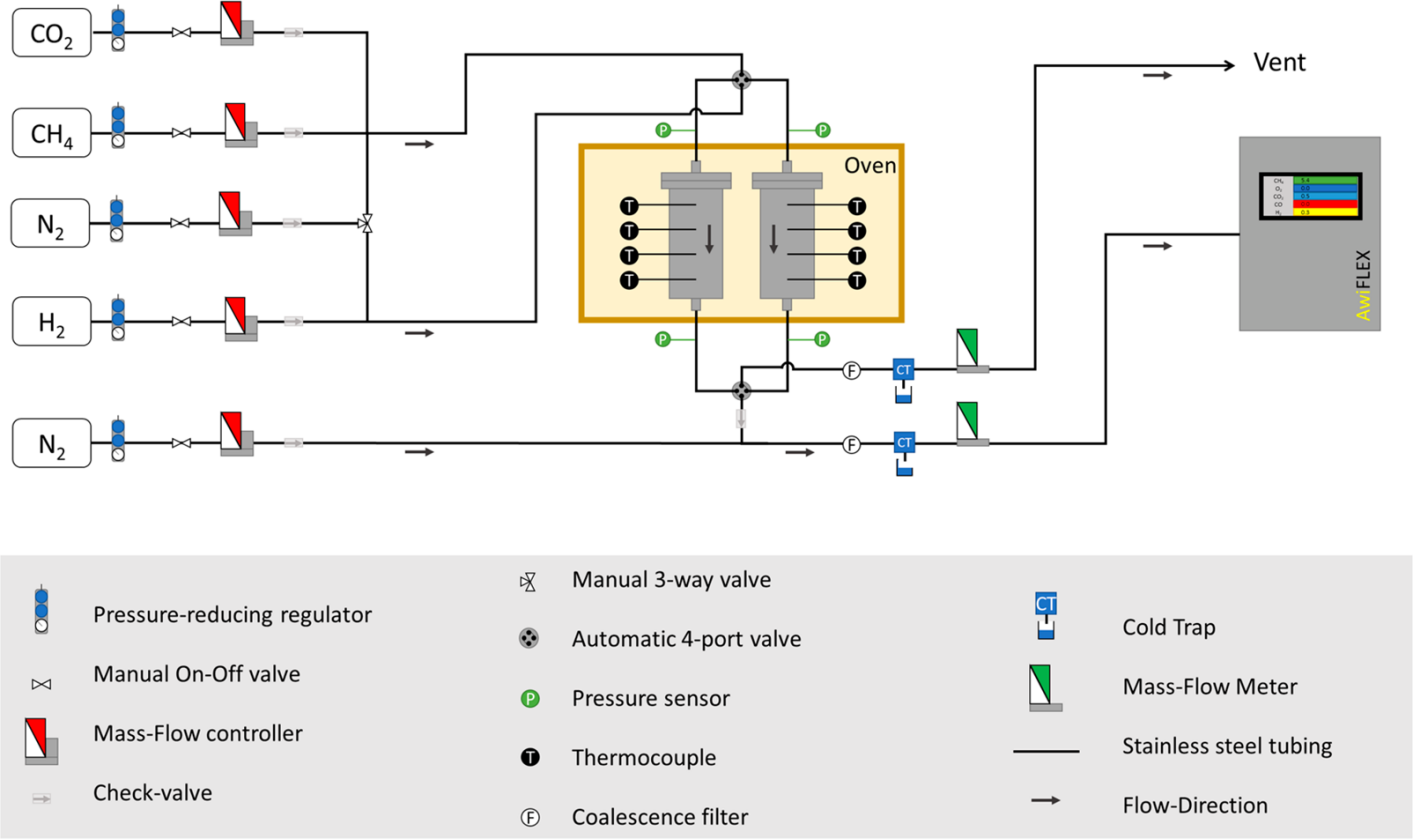
Experimental setup scheme.

A scheme of the described experimental setup is presented in Figure 2. The sorptive reactors (A and B) were placed inside a forced air convection oven (SNOL, Model 58/350), ensuring a homogeneous temperature distribution during the full process. The temperature of each reactor was measured through four type-K thermocouples, placed in contact with the bed (each thermocouple aligned with one of the top four catalyst layers). Mass flow controllers (Model F201CV from Bronkhorst-High Tech) were used to feed CO_2_ (99.8%, Air Liquide), H_2_ (99.999%, Air Liquide), N_2_ (99.999%, Air Liquide), and CH_4_ (99.995%, Air Liquide). The mass flow rate of the outlet streams was measured with two mass flow meters (Model F111B from Bronkhorst-High Tech) and corrected on the basis of their composition. Four pressure transducers (Model PX40C from OMEGA Engineering Inc.) were used to measure the total pressure at the entrance and outlet of the reactors. During the experiments, the water produced in the CO_2_ methanation reaction and present in the outlet stream was condensed and removed by a cold trap. The composition of the outlet stream was measured continuously using a gas analyzer from AWITE, Bioenergy GmbH, Model AwiFLEX Cool+. N_2_ was used to make up the analyzed stream and maintain the flow rate above the minimum value required by the gas analyzer; the influence of this additional inert stream has been deducted from the presented results. Two automated four-port valves (Model EUDA 3C4UWE by VICI, Valco Instruments Co. Inc.) were used to switch the inlet streams of the reactors (CO_2_ and CH_4_ during the sorption stage or H_2_ during the reactive regeneration) and to switch the outlet stream (from sorptive reactor A or B) forwarded to the analyzer.

#### Process Indicators

The performance of the cyclic sorption-reaction process was assessed through different indicators, namely, carbon dioxide sorption capacity, *q*_CO_2__ (eq 2), carbon dioxide conversion, *X*_CO_2__ (eq 3), methane productivity, Prod_CH_4__ (eq 4), and the number of moles of hydrogen fed per mole of methane produced,  (eq 6). Equations 7–9 were used to calculate the average outlet fraction of each component during the sorption stage, *y*_*i*_^OUT,S^ (*i* standing for CH_4_, CO_2_, H_2_, or CO), the average outlet fraction of each component *i* during the reactive regeneration stage, *y*_*i*_^OUT,RR^, and the average outlet fraction of component *i* during a full cycle (sorption and reactive regeneration stages), *y*_*i*_^OUT^, respectively. All calculations were made on a dry basis.
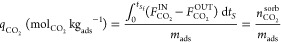
2

In eq 2, *F*_CO_2__^IN^ and *F*_CO_2__^OUT^ are the inlet and outlet molar flow rates of CO_2_, respectively, *t*_*S*_ is the time during the sorption stage, *t*_*S*_f__ is the duration of the sorption stage, *m*_ads_ is the sorbent mass and *n*_CO_2__^sorb^ is the number of moles of CO_2_ sorbed (i.e., retained inside the packed column) from those that are present in the feed.
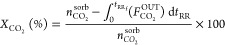
3

In eq 3, *t*_RR_ is the time during the reactive regeneration stage and *t*_RR_f__ is the duration of the reactive regeneration stage. The calculation of conversion with eq 3 is strictly valid for cyclic steady-state conditions.
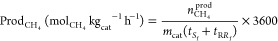
4

In eq 4, *n*_CH_4__^prod^ is the number of moles of CH_4_ produced by methanation, calculated according to eq 5, and *m*_cat_ is the catalyst mass.
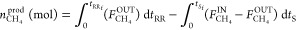
5

In eq 5, *F*_CH_4__^IN^ and *F*_CH_4__^OUT^ are the inlet and outlet molar flow rates of CH_4_, respectively.
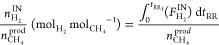
6

In eq 6, *n*_H_2__^IN^ is the number of moles of H_2_ that is fed to the reactor during the reactive regeneration stage, and *F*_H_2__^IN^ is the inlet molar flow rate of H_2_.
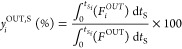
7

In eq 7, *F*_*i*_^OUT^ is the outlet molar flow rate of component *i* (CH_4_, CO_2_, H_2_, CO, or N_2_) and *F*^OUT^ is the outlet total molar flow rate.
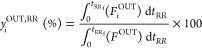
8
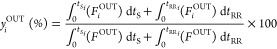
9

To evaluate the correctness of the experiments, the error of the carbon balance, Error_C_, was calculated according to eq 10.
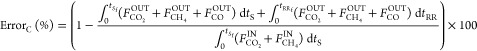
10

#### Experimental Procedure

Before the first experiment, the temperature of the packed reactors was increased to 350 °C, at a rate of 5 °C min^–1^, while feeding 200 mL_N_ min^–1^ of N_2_, followed by *in situ* catalyst reduction for 4 h at 350 °C, by feeding a mixture of 75 mL_N_ min^–1^ of H_2_ and 175 mL_N_ min^–1^ of N_2_ to each reactor. All the tests were performed at atmospheric pressure with a negligible pressure drop between the reactors’ inlet and outlet (<0.01 bar).

In each experiment, sorption–reactive regeneration cycles were performed on both reactors until a cyclic steady-state was reached. Each cycle, as indicated, consisted of two stages: a sorption stage during which the inlet stream was composed of CO_2_ and CH_4_ and a reactive regeneration stage during which the inlet was pure H_2_. To perpetuate the cyclic operation, the inlet streams (CO_2_/CH_4_ or H_2_) were periodically switched between the parallel reactors, so that when the sorptive reactor A was in the sorption stage the sorptive reactor B was in the reactive regeneration stage and vice versa. After each experiment, the reactors were left under an inert atmosphere, overnight.

In two final experiments of the parametric study, a 20 min purge stage was performed after the reactive regeneration stages. Thus, exceptionally in these tests, one cycle consisted of the sorption stage, reactive regeneration stage, and purge stage. During the purge stage, the inlet stream was composed of 200 mL_N_ min^–1^ of N_2_.

The parametric study aimed to assess the influence of different operating conditions on the cyclic sorption reaction process, namely the inlet CO_2_ content (study a) and the inlet flow rate (study b) during the sorption stage, the inlet flow rate during the reactive regeneration stage (study c), the stage duration (study d), the temperature (study e), and the inclusion of a purge stage (study f). Table 1 presents the operating conditions under which the tests were performed, with values highlighted in boldface type those referring to the parameter under study and that differ from the run used as reference. To verify that the stability of the materials used (catalyst and sorbent) was not compromised during the parametric study, the experiment described as reference experiment in Table 1 was performed periodically (ca. every 6 experiments); the relative error among experiments for all process indicators was <2%.

**Table 1:** Operating Conditions of the Parametric Study

	inlet CO_2_ content during sorption stage (%)	inlet flow rate during sorption stage (mL_N_ min^–1^)	inlet flow rate during reactive regeneration stage (mL_N_ min^–1^)	stage duration (min)	temperature (°C)	purge
reference experiment	50	75	100	20	350	no
study a	**30, 40**, 50, **60**	75	100	20	350	no
study b	50	**25, 50**, 75, **100**	100	20	350	no
study c	50	75	**50**, 100, **150, 200**	20	350	no
study d	50	75	100	**10**, 20, **30**	350	no
study e	50	75	100	20	**250, 300**, 350	no
study f	50	75	50, 100	20	350	**yes**

### Direct Hydrogenation

Additional experiments were performed to compare the proposed technology for biogas upgrading and valorization to one of the alternatives, namely, direct hydrogenation. Direct hydrogenation consists of simultaneously feeding the reactants (biogas and hydrogen) to a traditional fixed bed reactor.

For these tests, the reactive mixture composed of CH_4_, CO_2_, and H_2_ was fed directly to one of the previously described sorptive reactors (filled with an identical packed bed), in the described experimental setup. The mixture was continuously fed for over 120 min, a period long enough for the sorbent to be completely saturated with CO_2_, leaving the reactor functioning as a traditional fixed bed reactor and reaching a steady-state. An additional experiment was performed in which it was confirmed that the hydrotalcite does not catalyze the methanation reaction (data not shown).

To facilitate a comparison between the cyclic unit with the direct hydrogenation, the performance of the latter was assessed by CO_2_ conversion, *X*_CO_2__^DH^ (eq 11), CH_4_ productivity, Prod_CH_4__^DH^ (eq 12), moles of H_2_ fed per mole of CH_4_ produced,  (eq 13), and CH_4_ purity, *y*_CH_4__^OUT,DH^ (eq 14).
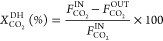
11


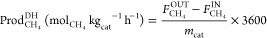
12


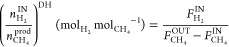
13


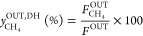
14


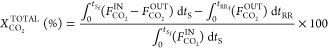
15

In eq 11, the CO_2_ conversion in the direct hydrogenation experiments (*X*_CO_2__^DH^) was calculated as a fraction of the total fed CO_2_ (the denominator), not as a fraction of the sorbed CO_2_, as in eq 3. Hence, an additional type of CO_2_ conversion was defined for the cyclic process (*X*_CO_2__^TOTAL^). This process indicator, calculated according to eq 15, allows a comparison between the direct hydrogenation and cyclic process, because it presents the CO_2_ conversion of the cyclic process as a fraction of the total fed CO_2_, just as in eq 11.

Just like the other process indicators defined in this section (eqs 11–14), *X*_CO_2__^TOTAL^ was only used in the Unit Optimization, Direct Hydrogenation and Future Work section, when the proposed cyclic process was compared to the direct hydrogenation, not in the parametric study. In this section, the cyclic process was also compared to the case of direct hydrogenation in thermodynamic equilibrium, which was simulated by employing the nonstoichiometric Gibbs free energy minimization method with Aspen Plus V12 software.

The experiments from the parametric study that were recreated with the direct hydrogenation method were the reference experiment, the experiment from study b that was performed with *Q*^IN,S^ = 50 mL_N_ min^–1^, and the experiment from study e that was performed at 300 °C. In the direct hydrogenation tests, the streams that, with the cyclic method, were fed alternately (biogas or H_2_), were now fed simultaneously (biogas and H_2_). The inlet flow rate of each species was maintained. Additionally, a final test was performed under conditions that had not been used in the parametric study: simulated biogas with a CO_2_ content of 30% and a total flow rate of 125 mL_N_ min^–1^, H_2_ inlet flow rate of 150 mL_N_ min^–1^, 5 min of stage duration, and a temperature of 250 °C.

## Results and Discussion

### Parametric Study

#### Reference Experiment

**Figure 3:**
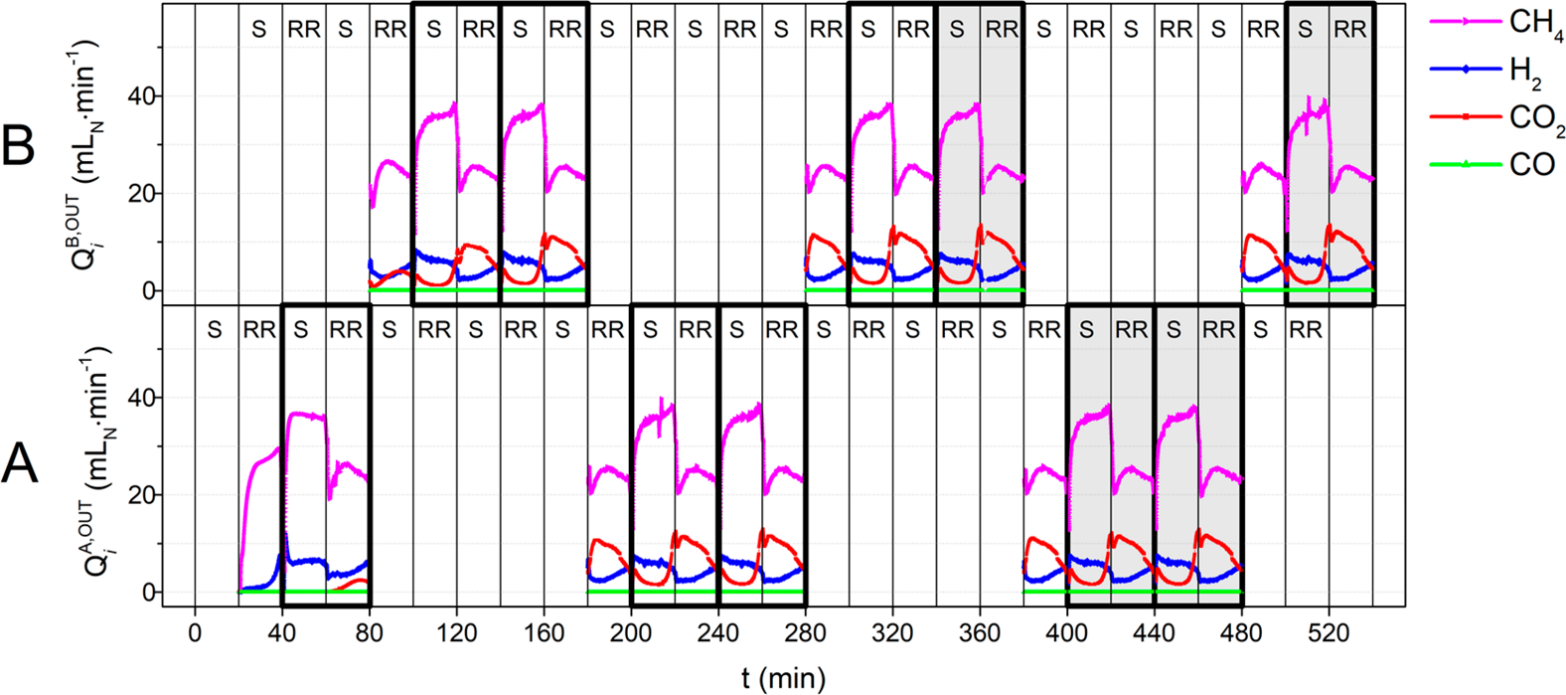
Partial flow rate of the outlet streams of sorptive reactor A (*Q*_*i*_^A,OUT^) and sorptive reactor B (*Q*_*i*_^B,OUT^) during the reference experiment (experimental conditions are given in Table 1). S and RR above each stage indicate whether it is a sorption (S) or reactive regeneration (RR) stage. The rectangles with bold lines highlight the sorption–reactive regeneration cycles for which the process indicators were calculated. The S/RR cycles that are shaded in gray correspond to a cyclic steady-state operation.

Figure 3 plots the outlet flow rate of each species during the reference experiment in both reactors (see Table 1). The cycles were run continuously in the two reactors (operating at 180° out of phase) for the full length of the experiment, yet the analyzer allowed measuring only one stream at a time, which explains the absence of data during some stages (i.e., the empty rectangles in Figure 3 for either reactor A or reactor B).

Figure 3 shows that the methane flow rate during the sorption stage is increasing over time and CO_2_ is being retained in the column (i.e., minimal outlet flow rate) until it breaks through near the end of the stage duration (20 min in this case). During this stage, there is also H_2_ in the outlet stream. The H_2_ detected at the beginning of the sorption stages is H_2_ that remained inside the column from the previous reactive regeneration stage, i.e., in the gas phase (and possibly adsorbed by the catalyst), and that is being purged.^36,37^ However, the majority of the H_2_ exiting the unit during the sorption stages results from the steam reforming of methane (SRM, in eq 16) followed by the water-gas shift reaction (WGS, in eq 17).
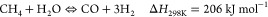
16
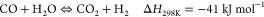
17

Hence, the CH_4_ fed in the sorption stage reacts with H_2_O captured in the sorbent during the previous reactive regeneration, forming CO (not detected in this experiment) that further reacts with H_2_O, forming CO_2_ and more H_2_. The sorption of H_2_O by hydrotalcite-based materials, and specifically by the sorbent used herein (PURAL MG30K by SASOL), has been observed and reported in the literature.^33,38,39^ The SRM is usually carried out at high temperatures (above 500 °C) in order to obtain the desired CH_4_ conversions.^40^ Nevertheless, as proven in an experiment described in the Supporting Information and whose results are presented in Figure S.1, it is possible for the SRM to occur at 350 °C, even if to a very low extent. As discussed in the Supporting Information, the overall stoichiometry of the SRM followed by WGS dictates that the consumption of 1 mol of CH_4_ produces 4 mol of H_2_. With regard to the reference experiment, this means that, even if these reactions occur to a low extent during the sorption stage, the effect of the H_2_ formed on the purity of the outlet stream may be significant, which explains the considerable outlet flow rate of H_2_ during the sorption stages (cf. blue line in S stages in Figure 3). The occurrence of SRM is also consistent with the increasing tendency of the outlet flow rate of CH_4_ during sorption stages (cf. Figure 3) and the inverse trend of H_2_. As the finite amount of H_2_O captured in the sorbent during the reactive regeneration stage is progressively consumed, the extent of the SRM starts decreasing and the outlet flow rate of CH_4_ approaches its inlet value.

During the regeneration stage, the methane flow rate suddenly decreases but later starts increasing when the previously retained CO_2_ starts being desorbed and converted to methane. The H_2_O molecules produced during this stage, through the Sabatier reaction, are also captured in the sorbent, actuating as a sorption-enhanced reactor and positively affecting methane formation, by shifting the reaction equilibrium toward the production of more CH_4_.^34^ Part of the CO_2_ is simply desorbed and exits the sorptive reactor unconverted. At the end of the reactive regeneration stages, the flow rate of methane starts decreasing again, as the CO_2_ inside the reactors and available for reaction progressively decreases. Simultaneously the flow rate of H_2_ at the outlet of the sorptive reactors starts increasing.

**Figure 4:**
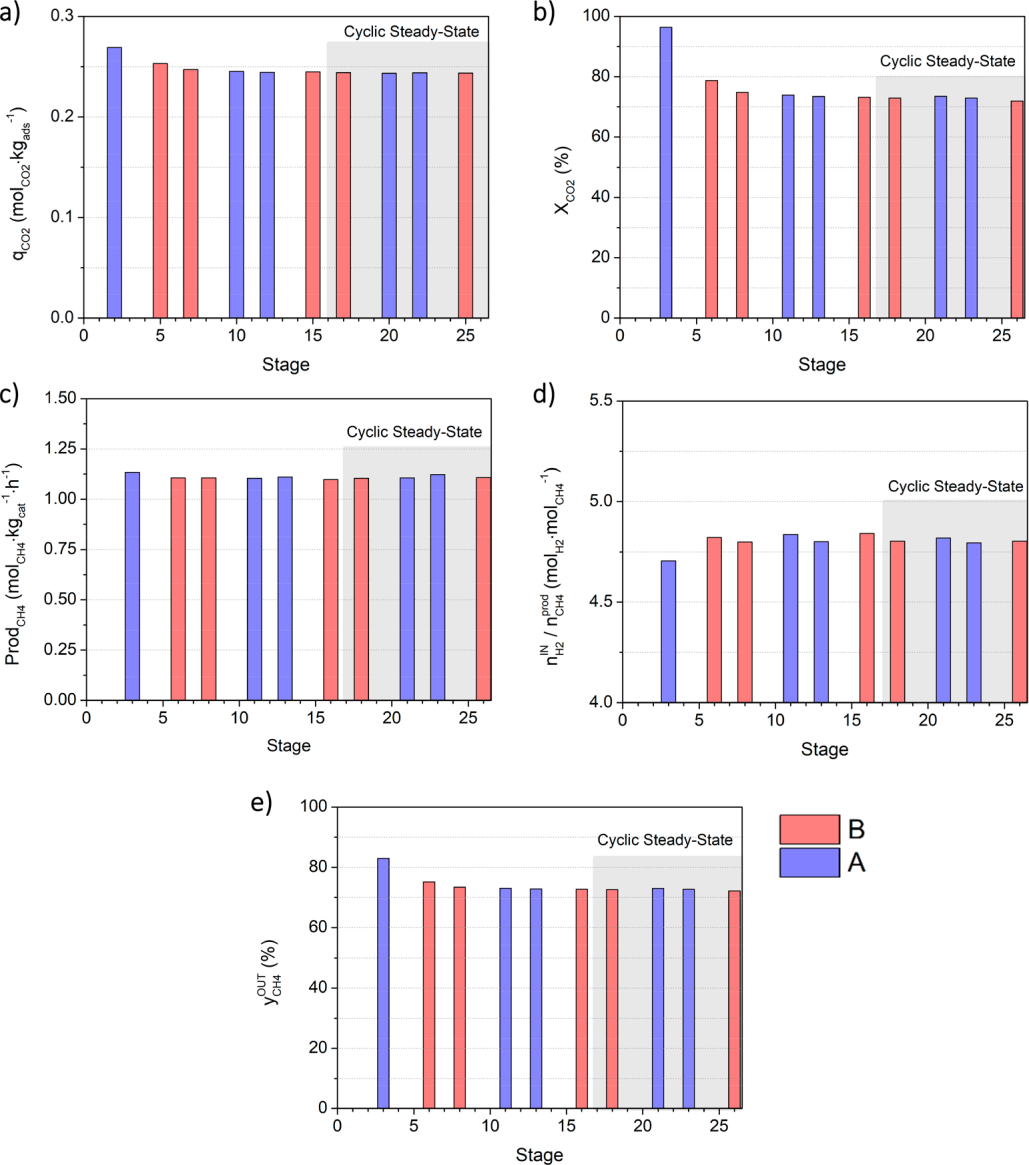
Process indicators calculated for the S/RR cycles measured in both sorptive reactors (A and B) during the reference experiment (experimental conditions are given in Table 1): (a) CO_2_ sorption capacity; (b) CO_2_ conversion; (c) CH_4_ productivity; (d) moles of H_2_ fed per mole of CH_4_ produced; (e) average outlet fraction of CH_4_ during a full cycle.

The rectangles with bold lines in Figure 3 enclose the cycles for which the process indicators were calculated. These indicators are presented in Figure 4. Such process indicators are important parameters for process optimization; although CO_2_ capture and its conversion are certainly important outputs, the assessment of the overall performance should also take into account the CH_4_ productivity and purity, as well as the amount of H_2_ fed per mole of CH_4_ produced—from the economic point of view, such ratio should not be above the stoichiometric value of 4 mol_H_2__ mol_CH_4__^–1^, given the constraints associated with the price of renewable-based H_2_.^7,41^

Figure 4a shows that the CO_2_ sorption capacity obtained at stage 2 was 0.269 mol_CO_2__ kg_ads_^–1^ and decreased in the following cycles until it reached a plateau (i.e., 0.244 mol_CO_2__ kg_ads_^–1^) from stage 15 onward. The sorption capacity loss of hydrotalcite-based materials with cycles is well-known in the literature, especially if the CO_2_-containing stream is dry and the regeneration stream consists of pure N_2_.^38,42^ Regeneration with steam is more effective and faster.^42,43^ This has been justified by the existence of an exchange site that can adsorb CO_2_ or H_2_O, in addition to two independent adsorption sites (one for CO_2_ and one for H_2_O). In this adsorption site, one sorbate species replaces the other if the partial pressure is altered, and so, if CO_2_ is sorbed, it can only be regenerated by H_2_O adsorption, not by N_2_ flushing.^39,44,45^ The present sorptive reactor concept benefits from steam being produced *in situ* as a product of the methanation reaction during the reactive regeneration, with additional costs for its production being avoided.^34^

Similarly, the other process indicators also varied in the initial phase of operation and reached a cyclic steady state after ca. stage 15 (i.e., at *t* = 340 min) (gray shaded area in Figure 4b–e). Hence, in the reference experiment, the average CO_2_ sorption capacity after a cyclic steady-state is reached was 0.244 mol_CO_2__ kg_ads_^–1^, the CO_2_ conversion was 72.8%, the CH_4_ productivity was 1.11 mol_CH_4__ kg_cat_^–1^ h^–1^, the moles of hydrogen fed per mole of methane produced was slightly above the stoichiometric value (4.81 mol_H_2__ mol_CH_4__^–1^), and the average CH_4_ outlet purity during a full cycle was 72.7%.

In the following sections, the results of the parametric study and obtained process indicators are presented. Figures S.2 and S.4 in the Supporting Information show, for every experiment of studies a–e, the partial outlet flow rate and the bed temperature obtained during one S/RR cycle performed on the cyclic steady state. The error of the carbon balance (cf. eq 10) for the cyclic steady state was lower than 7% in all experiments.

#### Effect of the CO_2_ Inlet Content : Study a

**Figure 5:**
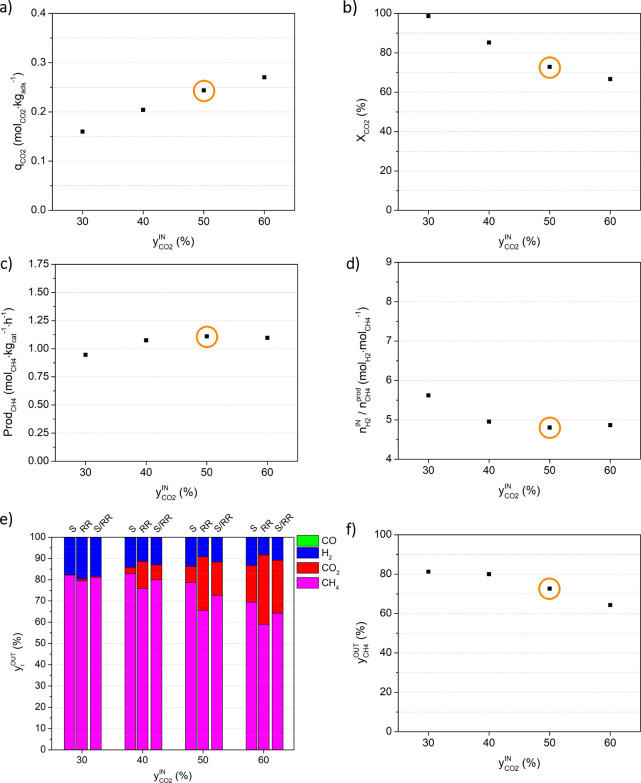
Effect of the inlet CO_2_ content (*y*_CO_2__^IN^) on the process indicators, namely (a) CO_2_ sorption capacity, (b) CO_2_ conversion, (c) methane productivity, (d) moles of hydrogen fed per mole of methane produced, (e) average outlet fraction of all components (CH_4_, CO_2_, H_2,_ and CO) during the sorption stage (S), reactive regeneration stage (RR) and full cycle (S/RR), and (f) average methane outlet fraction during a full cycle. Experimental conditions are given in Table 1. Orange circles mark the reference experiment.

Figure 5 plots the effect of the CO_2_ inlet content (assessed by study a) on the different process indicators obtained under cyclic steady-state conditions. The values presented in this figure are also given in Table S.1 in the Supporting Information.

In Figure 5a it is possible to observe that the increase in the inlet CO_2_ content (*y*_CO_2__^ΙΝ^) from 30% to 60% resulted in an increment in the amount of CO_2_ captured (as expected, given the increased driving force), evidenced by an improvement in the sorption capacity from 0.160 to 0.270 mol_CO_2__ kg_ads_^–1^, respectively. The enhancement of the sorption capacity was slightly more noticeable between experiments with lower contents of CO_2_ in the feed. This can be related to the fact that, as the CO_2_ inlet content is increased (namely for 50 and 60%), the sorbent gets closer to its full CO_2_ sorption capacity, leading to some of the fed CO_2_ passing through the sorptive reactor and not being sorbed (cf. the outlet CO_2_ fraction during this stage in Figure 5e).

Figure 5b presents the effect of the feed CO_2_ content in the conversion of sorbed CO_2_, *X*_CO_2__. No carbon monoxide was detected in either of the referred experiments, and so, the conversion of CO_2_ was solely related to CH_4_ formation. From the observation of Figure 5b it is possible to conclude that the conversion decreased with the rise of the CO_2_ inlet content. The reason for this tendency is that, as was mentioned, the amount of CO_2_ that was sorbed (and thus available to react) increased with *y*_CO_2__^IN^, but the amount of H_2_ that was fed during the reactive regeneration was kept constant (100 mL_N_ min^–1^). For higher feed CO_2_ contents there was too much CO_2_ for the fed H_2_ (especially considering that the stoichiometric H_2_/CO_2_ ratio is 4). Thus, there was some CO_2_ that was desorbed and left the reactor bed, unconverted. This is consistent with the fact that, as shown in Figure 5e, for CO_2_ contents in the feed of 40%, 50%, and 60%, the outlet CO_2_ content (red bars) during the reactive regeneration stage increased, while the outlet H_2_ content (blue) in the same stage (RR) was low and almost constant.

For an assessment of CH_4_ productivity, presented in Figure 5c, both process indicators already discussed are relevant: the amount of CO_2_ sorbed (reflected in *q*_CO_2__) increased with the CO_2_ inlet content, but the fraction of the sorbed CO_2_ that is converted into CH_4_ decreased and so, the CH_4_ productivity was not drastically altered.

Figure 5d presents the ratio between the H_2_ consumed and CH_4_ produced, . The amount of H_2_ fed was the same in all experiments but, as was discussed, the amount of produced CH_4_ (measured by productivity) was lower for the CO_2_ inlet content of 30%, and so the  ratio was higher (5.62 mol_H_2__ mol_CH_4__^–1^). For the remaining values of feed CO_2_ levels, the productivity was similar, and so the amount of H_2_ fed per mole of CH_4_ produced was nearly constant.

From the observation of Figure 5f it is possible to conclude that increasing the feed CO_2_ content caused a loss of CH_4_ purity. An analysis of Figure 5e) shows that the loss of CH_4_ purity was caused, essentially, by an increase in the CO_2_ outlet fraction in both the sorption (S) and reactive regeneration (RR) stages, for the reasons discussed above.

For this series of experiments, the maximum bed temperature variation was 7.79 °C and was registered for the experiment with the highest CO_2_ inlet content (Figure S.3a)). Since the CH_4_ production (a result of the exothermic Sabatier reaction) was similar for the experiments with *y*_CO_2__^IN^ values of 40%, 50%, and 60%, the difference in maximum temperature variation is presumably explained by the higher extent of adsorption with a CO_2_ inlet content of 60% (increased CO_2_ sorption capacity), which, overall, is also an exothermic phenomenon (cf. Figure S.4).

#### Effect of the Inlet Flow Rate during the Sorption Stage: Study b

**Figure 6:**
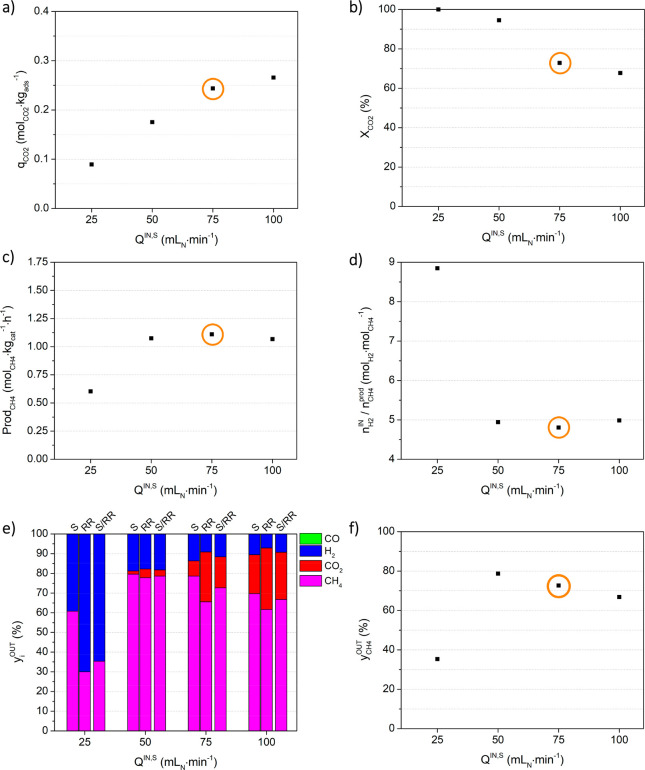
Effect of the inlet flow rate during the sorption stage (*Q*^IN,S^) on process indicators, namely (a) CO_2_ sorption capacity, (b) CO_2_ conversion, (c) methane productivity, (d) moles of hydrogen fed per mole of methane produced, (e) average outlet fraction of all components (CH_4_, CO_2_, H_2,_ and CO) during the sorption stage (S), reactive regeneration stage (RR), and the full cycle (S/RR), and f) average methane outlet fraction during the full cycle. Experimental conditions are given in Table 1. Orange circles mark the reference experiment.

Figure 6 presents the process indicators obtained during study b, i.e., with different inlet flow rates during the sorption stage (*Q*^IN,S^). Table S.2 lists the values presented in Figure 6. The variation of the inlet flow rate during the sorption stage had an effect similar to the variation of the CO_2_ inlet content (discussed in the previous section), which was expected since they both relate to the amount of CO_2_ being fed to the system. Hence, the amount of CO_2_ sorbed increased with the inlet flow rate, while the conversion of captured CO_2_ consistently decreased, resulting in a productivity of ca. 1.07 mol_CH_4__ kg_cat_^–1^·h^–1^ for all inlet flow rates during the sorption stage above 50 mL_N_ min^–1^. With regard to the CH_4_ purity, an optimum value (78.6%) was reached with a *Q*^IN,S^ value of 50 mL_N_ min^–1^, after which a further raise of the inlet flow rate resulted in an increased CO_2_ outlet content, i.e., CO_2_ breakthrough and waste (cf. Figure S.2b)). As presented in Figure S.3b), and for the same reasons described in the previous section, the maximum bed temperature variation followed the tendency of CO_2_ sorption capacity and reached its highest value (7.36 °C) when the inlet flow rate during sorption was 100 mL_N_ min^–1^.

Nevertheless, and unlike the experiments presented in the previous section, the presence of CO was detected, concretely in the experiment performed with an inlet flow rate of 100 mL_N_ min^–1^. In this experiment, the average outlet content of this undesired product was 298 ppm if the full cycle (S/RR) was taken into consideration, which is below European standards for biomethane injection into the gas grid, <1000 ppm.^46,47^ As the amount of CO produced was minimal, its weight on CO_2_ conversion was less than 0.1%. The CO was formed at the end of the sorption stage. While testing dual-function materials, Kosaka et al. also verified the presence of CO in the CO_2_ adsorption stages of experiments consisting of CO_2_ capture from a CO_2_/N_2_ mixture, followed by an N_2_ purge and finally CO_2_ methanation through the feeding of H_2_.^48^ Through the characterization of the tested materials, the authors concluded that the formation of CO during the adsorption step was related to the reaction of H_2_ previously adsorbed on the catalyst with the fed CO_2_. In a work by Miguel et al., the formation of CO was attributed to the reverse water-gas shift reaction (reverse reaction of eq 17), although its production was observed in a different step of the process, the reactive regeneration.^34^ As was stated, in the present work CO was only detected for the highest biogas inlet flow rate, and at the end of the sorption stage, after breakthrough, when the CO_2_ outlet flow rate was high (cf. Figure S.2b). Therefore, the detected CO was presumably formed by the steam reforming of methane (eq 16), and due to the high concentration of CO_2_ (after breakthrough), it was not further converted by the water-gas shift reaction (eq 17).

In conclusion, the use of lower feed biogas flow rates allowed avoiding the breakthrough of CO_2_ during the sorption stage, which was desirable not only because it reduced CO_2_ waste and outlet content during that stage but also because it mitigated the production of CO.

#### Effect of the Inlet Flow Rate during the Reactive Regeneration Stage: Study c

**Figure 7:**
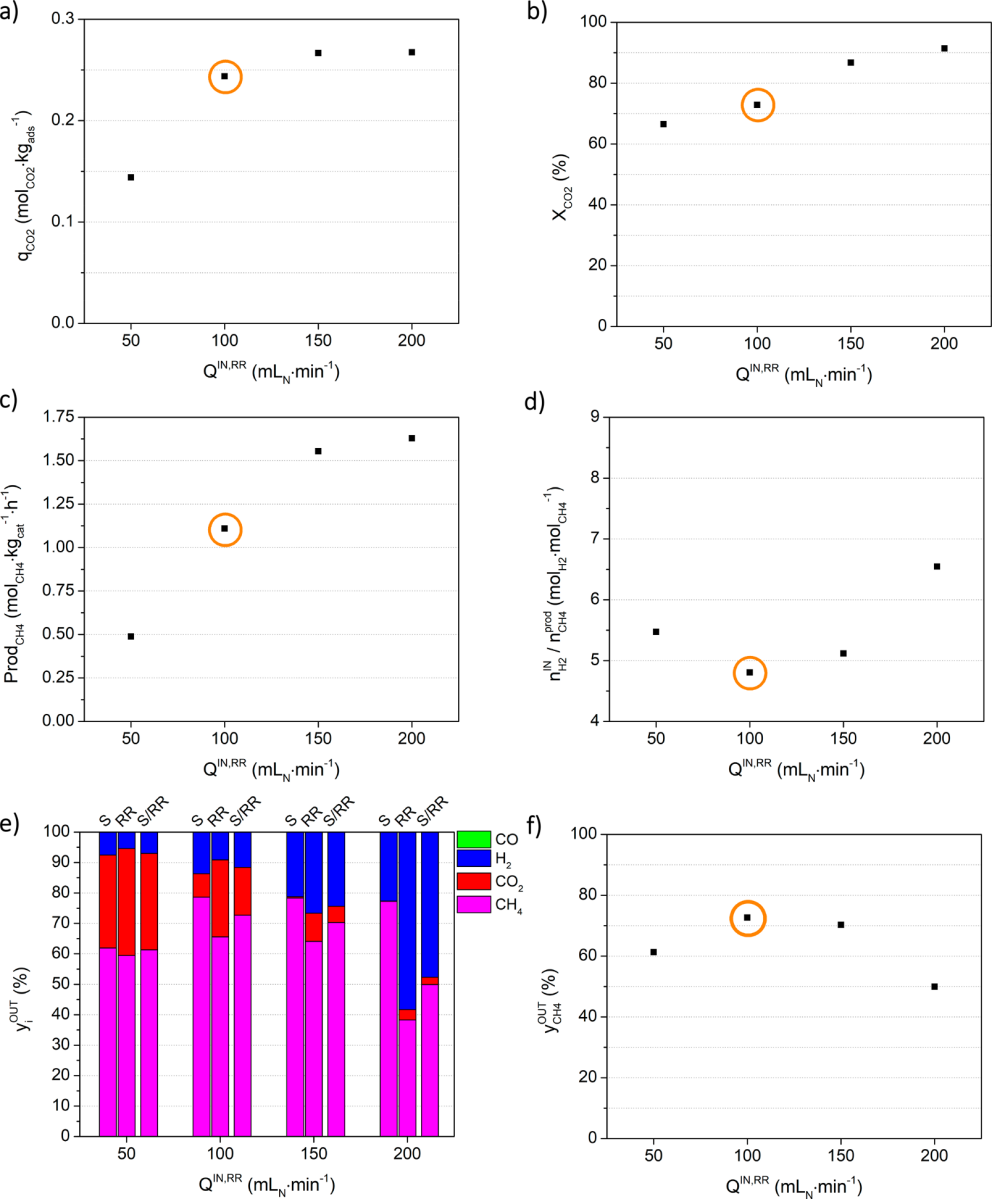
Effect of the H_2_ inlet flow rate during the reactive regeneration stage (*Q*^IN,RR^) on process indicators, namely (a) CO_2_ sorption capacity, (b) CO_2_ conversion, (c) methane productivity, (d) moles of hydrogen fed per mole of methane produced, (e) average outlet fraction of all components (CH_4_, CO_2_, H_2_, and CO) during the sorption stage (S), reactive regeneration stage (RR) and the full cycle (S/RR), and (f) average methane outlet fraction during the full cycle. Experimental conditions are given in Table 1. Orange circles mark the reference experiment.

Figure 7 depicts the influence of the H_2_ flow rate during the reactive regeneration stage (*Q*^IN,RR^) in the process indicators, assessed during study c. For the values presented in Figure 7, please refer to Table S.3. When the H_2_ flow rate was lower or higher than 100 mL_N_ min^–1^ (i.e., the reference experiment), the CO_2_ sorption capacity decreased or increased, respectively (cf. Figure 7a). Moreover, for an inlet flow rate equal or lower than 100 mL_N_ min^–1^, there was a breakthrough of CO_2_ during the sorption stage, while for higher flow rates, the same was not observed (cf. Figure S.2c). The key to understanding why the H_2_ flow rate used in the reactive regeneration can influence the sorption capacity lies in an analysis of the CO_2_ conversion and methane productivity (Figure 7b,c). The results show that both CO_2_ conversion and CH_4_ productivity increased with the H_2_ flow rate, as expected, which allows concluding that the effect of the H_2_ flow rate on sorption capacity is due to the better sorbent regeneration, which is also promoted by the highest amount of steam produced by the reaction (which is beneficial for the reasons already presented in the Reference Experiment section). Such an enhancement of the sorption capacity, CO_2_ conversion, and CH_4_ production was noticeable when the H_2_ flow rate was changed from 100 to 150 mL_N_ min^–1^. However, for a flow rate of 200 mL_N_ min^–1^ a substantial dilution of the outlet stream with unreacted H_2_ occurred, severely affecting the methane purity (i.e., from 73.3% to 49.9%) and increasing the amount of H_2_ wasted, represented by the  ratio.

The maximum bed temperature variation (8.17 °C, Figure S.3c) was observed for the experiment with a *Q*^IN,RR^ value of 100 mL_N_ min^–1^, in which CO_2_ sorption and CO_2_ methanation were both maximized.

In the experiment performed with the lowest H_2_ inlet flow rate, i.e., a *Q*^IN,RR^ value of 50 mL_N_ min^–1^, there was the production of CO. The average CO content in the outlet stream during the full cycle (S/RR) was 392 ppm, again below the European specifications for biomethane injection into the grid.^46,47^ Once more, CO was only detected at the end of the sorption stage, in an experiment where there was a CO_2_ breakthrough (cf. Figure S.2c). Thus, the conclusions drawn in the previous section (with regard to the causes of CO formation) may also be applied to this experiment.

In summary, the results in this section highlight the need for precise H_2_ dosing to the reactor to enhance practically all process indicators, but without severely compromising methane purity and/or causing CO formation.

On comparison of the consequences of increasing the CO_2_ inlet (whether it is through a higher *y*_CO_2__^IN^ or *Q*^IN,S^) with increasing H_2_ inlet, it is possible to conclude that the effect on CO_2_ capacity was the same: a higher reactant inlet led to a higher CO_2_ capacity (albeit for distinct reasons). With regard to CO_2_ conversion, the same was not observed: a higher CO_2_ content and feed flow rate during the sorption resulted in lower conversion, while a higher flow rate during regeneration obtained enhanced CO_2_ conversions. Consequently an increase in CO_2_ content and feed flow rate during the sorption stage did not result in a considerable variation of the amount of CH_4_ produced, whereas a higher H_2_ inlet flow rate improved the CH_4_ productivity. This allows concluding that the lower conversion obtained with high CO_2_ inlet conditions (high *y*_CO_2__^IN^ and *Q*^IN^^,S^) was caused by a lack of H_2_ and not by low catalyst activity. The presence of H_2_ in the outlet streams during reactive regeneration in experiments with high *y*_CO_2__^IN^ and *Q*^IN^^,S^ values (cf. Figures 5e and 6e) was related to the reversible, and therefore thermodynamically limited, nature of the methanation reaction.

#### Effect of the Stage Duration: Study d

**Figure 8:**
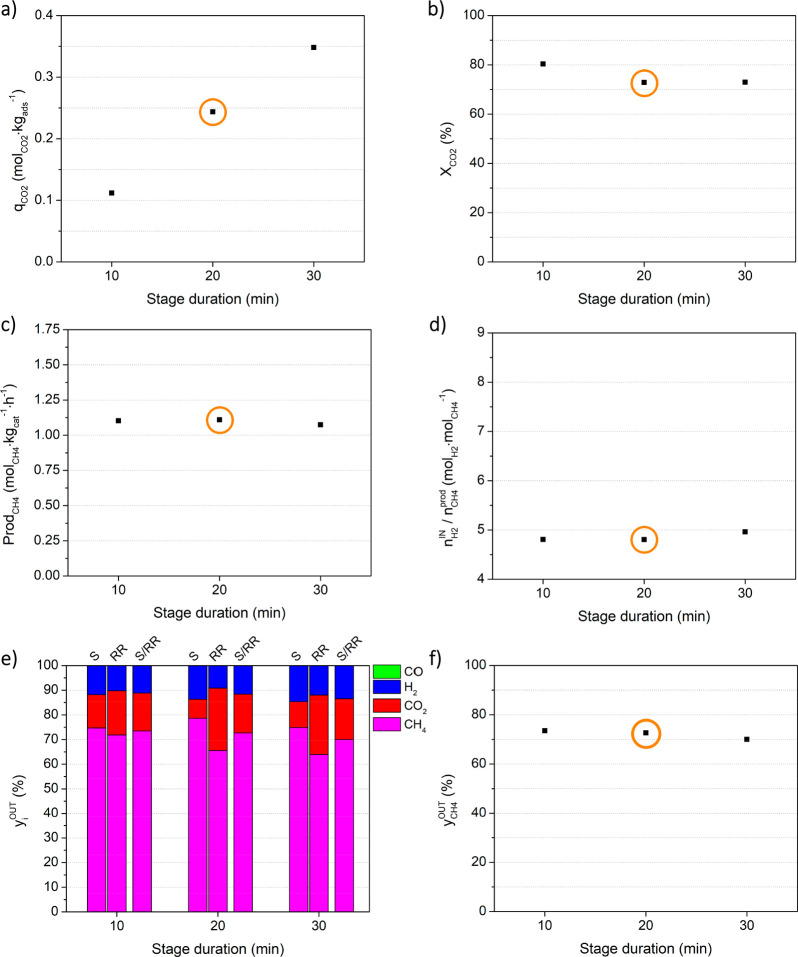
Effect of the stage duration on process indicators, namely (a) CO_2_ sorption capacity, (b) CO_2_ conversion, (c) methane productivity, (d) moles of hydrogen fed per mole of methane produced, (e) average outlet fraction of all components (CH_4_, CO_2_, H_2_, and CO) during the sorption stage (S), reactive regeneration stage (RR) and the full cycle (S/RR), and (f) average methane outlet fraction during the full cycle. Experimental conditions are given in Table 1. Orange circles mark the reference experiment.

Figure 8 presents the results of study d, with regard to the effect of the variation of stage duration, which is equal in both sorption and reactive regeneration. The results are also reported in Table S.4. In Figure 8a it is possible to observe that the amount of CO_2_ sorbed increased almost linearly with an extension of stage duration, i.e., the increase of stage duration from 10 to 20 and 30 min resulted in almost double and triple the amount of CO_2_ captured during the sorption stage, respectively. As the duration of the reactive regeneration stage, together with the sorption stage, was also doubled and tripled, the amount of CO_2_ converted to CH_4_ increased accordingly, resulting in a fraction of converted CO_2_ (or CO_2_ conversion) slightly higher for 10 min stages, but overall, this conversion was not largely affected by a variation of stage duration (cf. Figure 8b). In fact, the CH_4_ productivity remained nearly constant because the increase of methane produced was counterbalanced by the increase of the cycle duration to the same extent (please refer to eq 4).

The influence of stage duration on CO_2_ breakthrough can be explained by how efficiently the sorbent was regenerated: in the experiments with longer cycles, the breakthrough was observed later in the sorption stage (cf. Figure S.2d) because the reactive regeneration was prolonged and the number of active sites available for CO_2_ capture was improved.

The maximum bed temperature variation was ca. 7 °C for all experiments (as presented in Figure S.3d).

The presence of CO was detected at the end of the sorption stage of the experiment with 30 min stages, after the CO_2_ breakthrough (cf. Figure S.2d). The average CO outlet for S/RR was minimal, only 72 ppm. In sum, the performance of the sorptive reactors, CH_4_ productivity, and purity were not severely affected by the variation of stage duration, although for the 30 min stage there was the formation of CO, even if in minimal amounts.

#### Effect of Temperature: Study e

**Figure 9:**
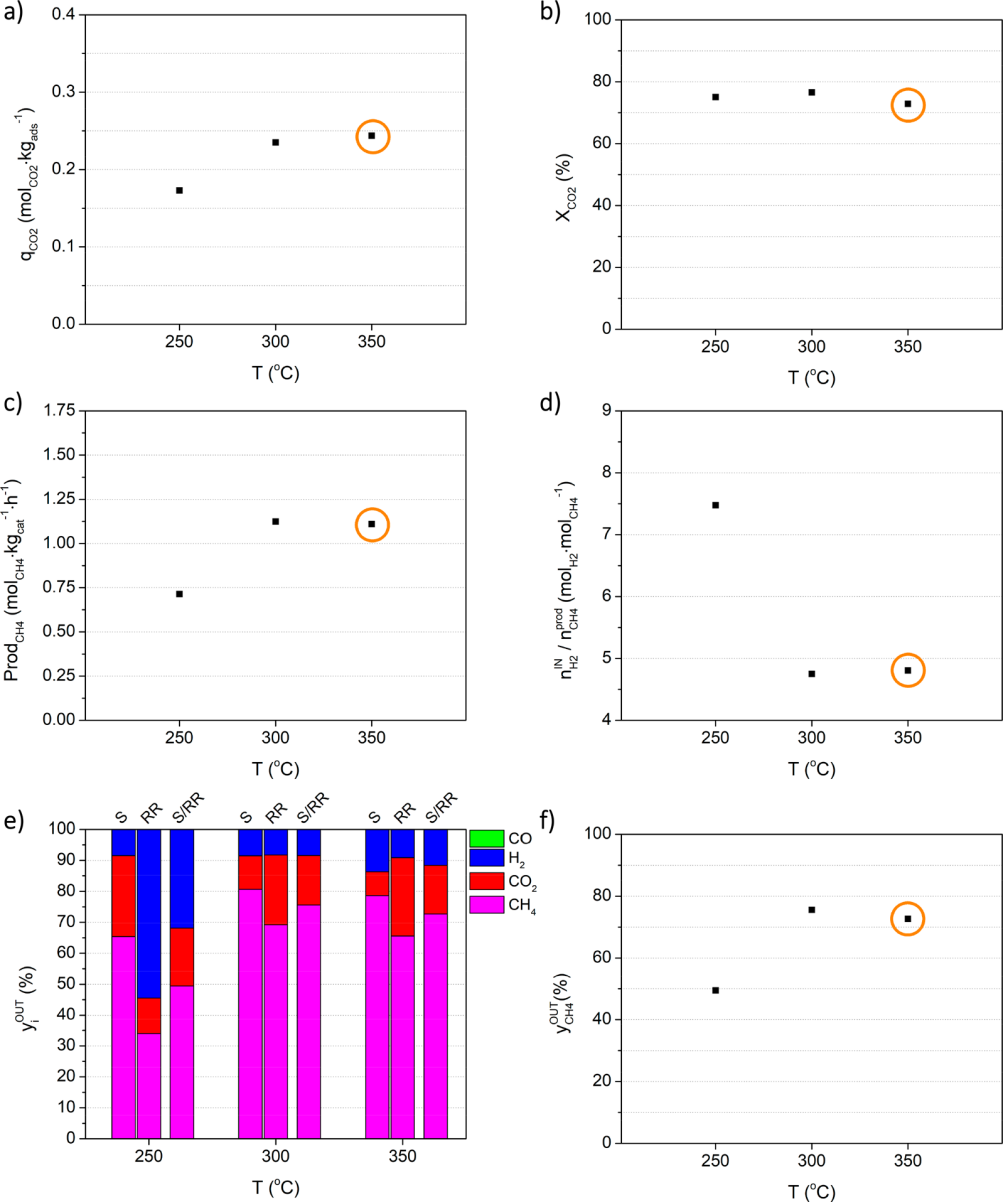
Effect of temperature on process indicators, namely (a) CO_2_ sorption capacity, (b) CO_2_ conversion, (c) methane productivity, (d) moles of hydrogen fed per mole of methane produced, (e) average outlet fraction of all components (CH_4_, CO_2_, H_2_, and CO) during the sorption stage (S), reactive regeneration stage (RR) and the full cycle (S/RR), and (f) average methane outlet fraction during the full cycle. Experimental conditions are given in Table 1. Orange circles mark the reference experiment.

Figure 9 depicts the effect of temperature (*T*) on the process indicators, assessed by study e. The corresponding results are also given in Table S.5. An increase in the CO_2_ sorption capacity with temperature, as shown in Figure 9a, has been reported for hydrotalcite-based materials at temperature ranges similar to those assessed herein.^34,38,50−54^

The methanation reaction is thermodynamically unfavored at high temperatures, which is consistent with the CO_2_ conversion obtained at 300 °C being slightly higher than that at 350 °C (Figure 9b). At low temperatures, the methanation of CO_2_ is kinetically hindered; however, the conversion of CO_2_ was not heavily affected by a decrease in temperature to 250 °C. This is because, at this temperature, the CO_2_ sorption capacity is lower and so, although the fraction of CO_2_ converted is similar, the total amount is lower (cf. lower productivity at 250 °C in Figure 9c). Overall, Figure 9 shows that, under the considered operating conditions, the best performance was obtained at 300 °C, since the CH_4_ purity decreased at 350 °C, and at 250 °C both the CH_4_ productivity and purity were lower.

Figure S.2e shows that operating at lower temperatures entails another advantage: at 250 and 300 °C, the outlet flow rate of H_2_ during the sorption stage is lower, which is consistent with the steam reforming of methane being both thermodynamically and kinetically hindered at lower temperatures.

With regard to the temperature in the reactor bed, the variation observed at 250 °C (Figure S.3e) was much lower than that at 300 and 350 °C, which was expected on consideration that, at 250 °C, the CO_2_ sorption capacity and CH_4_ productivity are considerably lower. As stated, at 300 and 350 °C the extents of CO_2_ sorption and conversion were similar, and yet the maximum temperature variation was more than 1 °C higher at 350 °C. Figure S.4e shows that the temperature variations measured along the reactor (by thermocouples 1–4, from closest to farthest from the inlet) are more similar at 300 °C in comparison to those at 350 °C. At 350 °C, during RR, the methanation reaction is more kinetically favored in comparison to that at 300 °C, and so more CO_2_ is converted at the beginning of the reactor, creating a high-temperature peak in the thermocouple closest to the inlet (T_1_), higher than that registered by T_4_. At 300 °C the kinetics are slower, and so the conversion of CO_2_ is not as localized at the beginning of the reactor but is more evenly distributed through all the catalyst layers (T_1_ is closest to T_4_). Hence, on comparison of T_4_, the variation at 300 °C is greater than that at 350 °C, but on comparison of T_1_ (in which the maximum temperature variation is inevitably read) the peak is ca. 1 °C higher.

Operating at lower temperatures with similar or enhanced performance is particularly relevant and is an advantage of this integrated process, since heat management and the associated safety requirements are the main issues with regard to the design and CAPEX/OPEX of conventional methanation reactors. In addition, as shown by Miguel et al., decreasing the operation temperature disfavors the endothermic reverse water-gas shift reaction and the formation of undesired CO, although the presence of a such compound was not observed in either of the experiments discussed in this section.^34^

#### Purge Stage: Study f

Study f assessed the addition of an N_2_ flushing step (purge stage) after the reactive regeneration stage. For reasons of brevity the results obtained and respective discussion are presented in the Supporting Information, in a section entitled Parametric Study–Purge Stage–Study f: namely, in Figures S.5 and S.6 and Tables S.6 and S.7.

In conclusion, it was observed that, under the tested conditions, the addition of a purge stage allowed a better regeneration of the sorbent (due to additional desorption of CO_2_ and H_2_O), resulting in an increase in CO_2_ sorption capacity and mitigating the steam reforming of CH_4_. However, the N_2_ flush negatively affected the productivity and led to the presence of N_2_ in the outlet stream of sorption stages, diluting the purified CH_4_ (and thus counterbalancing the positive effect of the inhibition of CH_4_ consumption through SRM followed by WGS).

### Unit Optimization, Direct Hydrogenation and Future Work

Table 2 presents, in summary, the operating conditions of the parametric study that resulted in the highest CH_4_ purity and productivity. These are the experiments performed with an inlet CO_2_ concentration of 30% from study a and with a feed flow rate during reactive regeneration of 200 mL_N_ min^–1^ from study c.

**Table 2:** Highest CH_4_ Productivity and Purity Obtained and Respective Operating Conditions

study	*y*_CO_2__^IN^ (%)	*Q*^IN,S^ (mL_N_ min^–1^)	*Q*^IN,RR^ (mL_N_ min^–1^)	stage duration (min)	*T* (°C)	Prod_CH_4__ (mol_CH_4__ kg_cat_^–1^ h^–1^)	*y*_CH_4__^OUT^ (%)
a	**30**	75	100	20	350	0.95	81.2
c	50	75	**200**	20	350	1.63	49.9

However, there were other experiments in which an interesting compromise between purity and productivity was obtained, such as the experiments performed with a feed CO_2_ concentration of 40% from study a (Prod_CH_4__ = 1.07 mol_CH_4__ kg_cat_^–1^ h^–1^ and *y*_CH_4__^OUT^ = 80.0%), the experiment with a hydrogen flow rate of 50 mL_N_ min^–1^ from study c (Prod_CH_4__ = 1.55 mol_CH_4__ kg_cat_^–1^ h^–1^ and *y*_CH_4__^OUT^ = 70.3%), and the test at 300 °C from study e (*Prod*_CH_4__ = 1.12 mol_CH4_·kg_cat_^–1^·h^–1^ and *y*_CH_4__^*OUT*^ = 72.7%).

To better understand in which conditions the cyclic approach would be advantageous, four additional tests were performed using direct hydrogenation. The results obtained in these experiments are presented in Table 3, alongside the respective operating conditions. In Table 3, each experiment performed (numbered 1–4) has three associated methods: the cyclic method (the technology proposed in this work, CY), direct hydrogenation (DH), wherein only one conventional packed bed reactor was employed, and the case of direct hydrogenation in thermodynamic equilibrium (TE). The performance indicators presented are the CO_2_ conversion (considering the total amount of CO_2_ fed), CH_4_ productivity, the ratio of H_2_ fed by CH_4_ produced, CH_4_ purity, and finally the maximum bed temperature variation.

**Table 3:** Operating Conditions and Process Indicators Obtained in the Test Numbers 1–4, Performed by Direct Hydrogenation (DH), and Comparison with the Results of the Cyclic Method (CY) and Thermodynamic Equilibrium (TE)

test no.	*Q*_CO_2__^IN^ (mL_N_ min^–1^)	*Q*_CH_4__^IN^ (mL_N_ min^–1^)	*Q*_*H*2_^IN^(mL_N_ min^–1^)	*T* (°C)	stage duration (min)	method	*X*_CO_2__^TOTAL^ (%)^a^	Prod_CH_4__ (mol_CH4_ kg_cat_^–1^ h^–1^)^b^	n_H_2__^IN^/*n*_CH_4__^prod^^c^ (mol_H_2__ mol_CH_4__^–1^)	*y*_CH_4__^OUT^ (%)^d^	Δ*T*_max_ (°C)
1	37.5	37.5	100	350	20	CY	66.4	1.11	4.81	72.7	7.1
						DH	59.2	2.20	4.84	72.1	9.3
						TE	60.7		4.42	71.2	
											
2	25	25	100	350	20	CY	92.9	1.07	4.94	78.7	6.3
						DH	87.6	2.14	4.99	75.1	9.6
						TE	88.3		4.54	76.1	
											
3	37.5	37.5	100	300	20	CY	67.2	1.12	4.75	75.6	6.0
						DH	62.5	2.96	4.69	75.9	8.7
						TE	63.7		4.20	77.0	
											
4	37.5	87.5	150	250	5	CY	75.2	1.02	7.79	66.2	3.4
						DH	57.2	1.91	8.37	59.0	5.2
						TE	96.0		4.17	95.5	

a CO_2_ conversion was calculated by eq 15 for the cyclic method (CY) and by eq 11 for direct hydrogenation (DH) and thermodynamic equilibrium (TE).

b CH_4_ productivity was calculated by eq 4 for CY and by eq 12 for DH and TE.

c H_2_ fed by CH_4_ produced ratio was calculated by eq 6 for CY and by eq 13 for DH and TE.

d CH_4_ purity was calculated by eq 9 for CY and by eq 14 for DH and TE.

From the analysis of tests 1–3 in Table 3 and from a comparison between the CY and DH methods, it is possible to conclude that, in these conditions, the use of the cyclic unit resulted only in a slight gain in the CO_2_ conversion. The improvement in these process indicators was not very significant, and given the error in carbon balance the use of the cyclic method is difficult to justify. Furthermore, since in the direct hydrogenation the inlet streams that would otherwise be fed alternately were fed simultaneously (to one single reactor), the methane productivity obtained with the DH method was higher than that obtained with the cyclic unit. An analysis of the process indicators obtained in thermodynamic equilibrium (TE) allows concluding that during the first three experiments the direct hydrogenation method was operating nearly at thermodynamic equilibrium. Even though the concept of “thermodynamic equilibrium” cannot be directly applied to the cyclic unit (given its dynamic nature), since the inlet streams are the same for DH and CY, it is possible to conclude that there was too much catalyst for the amount of CO_2_ available. This explains why the results obtained with the cyclic unit were similar to (or only slightly above) those obtained through direct hydrogenation (and those predicted by the thermodynamic equilibrium). With regard to the maximum bed temperature variation, also presented in Table 3, the use of the cyclic unit presented some advantages (because the exothermic methanation occurs simultaneously with the endothermic CO_2_ desorption), although the difference was only ca. 3 °C. The dilution of the catalyst in sorbent and inert spheres attenuates the bed temperature variations, and so this advantage would be strengthened if, as in the industrial case, the catalyst was not diluted.

An additional fourth experiment was conducted far from the thermodynamic equilibrium, under conditions that had not yet been tested in the parametric study. The operating conditions and process indicators obtained are also presented in Table 3. The partial outlet flow rate of each component at steady state is presented in Figure S.7, for both the CY and DH methods. Table 3 shows that, under these conditions, the CO_2_ conversion obtained with the cyclic unit was 75.2%, while with DH it was 57.2%. Also, the remaining process indicators (except for the CH_4_ productivity, for the reasons already stated) were enhanced in the fourth experiment, thus highlighting that the proposed cyclic unit presents clear advantages over the direct hydrogenation method, especially if the latter is working in conditions far from the thermodynamic equilibrium. Furthermore, the cyclic unit presents more versatility (generating two different outlet streams that can be mixed or not, instead of one) and it can reduce the risks associated with poor heat management. It is relevant to note that, even in the fourth experiment (in which the CY showed a greater advantage over DH), the cyclic unit was not optimized and so the potential of this method is much greater than that reported.

In conclusion, the experiments performed in this work not only resulted in the proof of concept of the continuous adsorption-reaction process for biomethane purification and production but also allowed important conclusions to be drawn with regard to future optimization: the fact that the sorption capacity is highly dependent on its regeneration (and on the H_2_O produced during methanation) and that the careful dosing of H_2_ during reactive regeneration is crucial and can avoid CO_2_ breakthrough during the sorption stage. Also, and for the same reason, the shortening of the cycle caused the breakthrough to occur earlier in the sorption stage, because a shorter reactive regeneration caused a less efficient regeneration of the sorbent. The CO_2_ breakthrough (which caused CO_2_ waste, loss of CH_4_ purity, and ultimately CO formation) was also avoided by the reduction of the inlet flow rate of biogas. The steam reforming of methane (and water-gas shift reaction) occurred during the sorption stage and affected the process, consuming CH_4_ and reducing its outlet purity. A reduction in the temperature allowed the mitigation of the steam reforming of methane, and at 300 °C the process indicators were not compromised. The addition of a purge stage after reactive regeneration also reduced the occurrence of the steam reforming of methane, due to the desorption of H_2_O during the purge. On the other hand, the purge stage led to the dilution of the outlet stream during the sorption stage in N_2_.

In the future, other variables should be studied aiming at the optimization of the cyclic method, increasing CH_4_ purity and productivity. The use of other catalysts (for example nickel-based) and sorbents, in other amounts, ratios, and bed configurations (e.g., mixed), should also be considered. The implementation of stages of different duration, for instance, shorter sorption steps (that avoid CO_2_ breakthrough) coupled with longer reactive regeneration stages, could also be beneficial. Similarly, an exploration of different purge durations, flow rates, or even methods may pose some advantages. The performance of longer experiments would also be relevant to assessing the stability of the materials (key for industrial-scale applications) and the resistance to impurities such as CO, which can be produced in the process and is known to cause the deactivation of some CO_2_ hydrogenation catalysts.^55^

## Conclusions

A novel method for continuous biogas upgrading and valorization of CO_2_ to CH_4_ was proposed and successfully tested. A parametric study was performed to assess the effect of six operating conditions, (a) the inlet CO_2_ content and (b) inlet flow rate during the sorption stage, (c) the inlet flow rate during the reactive regeneration stage, (d) the stage duration, (e) the temperature and also the effect of (f) an additional purge stage. Under the tested operating conditions, an increase in CO_2_ inlet fraction and inlet flow rate during the sorption stage had similar effects, leading to higher CO_2_ sorption capacity. This increase was not followed by a higher CO_2_ conversion, ultimately resulting in similar CH_4_ productivities and lower purity. On the other hand, the careful dosing of the H_2_ flow rate during reactive regeneration was proven to be very relevant to all process indicators. Up to a certain point (150 mL_N_ min^–1^), increasing the H_2_ flow rate had a very positive effect on the CO_2_ sorption capacity, CO_2_ conversion, and thus CH_4_ productivity, without severely compromising CH_4_ purity. Changing the stage duration did not affect considerably the unit performance, as the CH_4_ productivity and purity were both similar for 10, 20, and 30 min stages. With regard to the operating temperature, it was concluded that it is more advantageous to operate at 300 °C than at 350 °C because, in addition to slightly improving CH_4_ purity while maintaining productivity, it reduces risks associated with poor heat management and CO formation. Working at lower temperatures also allowed reducing the undesired steam reforming of methane that occurred during the sorption stage. The presence of CO was only detected when the highest inlet flow rate was used during the sorption stage (100 mL_N_ min^–1^), the lowest inlet flow rate was used during the reactive regeneration stage (50 mL_N_ min^–1^), and the longest stage duration was used (30 min). The inclusion of a purge stage after reactive regeneration caused the desorption of the remaining CO_2_ and H_2_O, which increased the amount of CO_2_ sorbed and reduced the steam reforming of CH_4_ during the sorption stage. Nevertheless, the presence of N_2_ in the sorption stage counterbalanced the positive effect, affecting CH_4_ purity. The productivity was also compromised by the addition of the purge stage.

A comparison of the cyclic method to direct hydrogenation allowed us to conclude that the former is more advantageous under conditions far from the thermodynamic equilibrium.

As was stated, the concept was proven and the parametric study allowed concluding that certain operating conditions can have a substantial effect on the performance of the cyclic unit and should be carefully considered for the optimization of the process. Still, other variables should also be studied in order to achieve higher CH_4_ productivity and purity and thus use the novel adsorption-reaction process for biomethane purification and production at its full potential.

## Supporting Information Available

Experiment on steam reforming of methane, outlet flow rates and bed temperature during one S/RR cycle performed on cyclic steady state, process indicators, and maximum bed temperature variation (at steady state) obtained in studies a–e of the parametric study, results obtained in study f (purge stage) and respective discussion, and partial outlet flow rate during direct hydrogenation and comparison to the cyclic method (PDF)
