# Supplementary material for: Novel Adsorption–Reaction Process for Biomethane Purification/Production and Renewable Energy Storage
Source: ACS Sustain Chem Eng. 2022 Jun 7;10(24):7833–51. doi: 10.1021/acssuschemeng.1c06844 (PMC9793493; doi:10.1021/acssuschemeng.1c06844)
Supplement: Supplementary file 1 — sc1c06844_si_001.pdf [file sc1c06844_si_001.pdf]

Supporting Information

Novel Adsorption-Reaction Process for Biomethane Purification/Production and Renewable Energy Storage

Joana A. Martins[a,c], Carlos V. Miguel[a,+], Alírio E. Rodrigues[b,c], Luis M. Madeira[a,c]*

[a]LEPABE, Laboratory for Process Engineering, Environment, Biotechnology and Energy, Chemical Engineering Department, Faculty of Engineering, University of Porto, Rua Dr. Roberto Frias, 4200-465 Porto, Portugal

[b]LSRE - LCM, Laboratory of Separation and Reaction Engineering - Laboratory of Catalysis and Materials, Chemical Engineering Department, Faculty of Engineering, University of Porto, Rua Dr. Roberto Frias s/n, 4200-465 Porto, Portugal

[c]ALiCE, Associate Laboratory in Chemical Engineering, Faculty of Engineering, University of Porto, Rua Dr. Roberto Frias, 4200-465 Porto, Portugal

[+]Present address: Fraunhofer Portugal AWAM - Research Center for Smart Agriculture and Water Management, Regia Douro Park - Parque de Ciência e Tecnologia, 5000-033 Vila Real, Portugal

*Email: mmadeira@fe.up.pt; Phone: +351 22 508 1519

Number of Pages: 14

Number of Figures: 7

Number of Tables: 7

Experiment on steam reforming of methane

To ascertain if the H_2 detected in the sorption stages was produced through the steam reforming of methane (Eq. 16) followed by water gas shift reaction (Eq. 17), *i.e.*, if the CH_4 present in the biogas was reacting with the H_2O sorbed in the hydrotalcite in the previous reactive regeneration stage, an experiment was conducted as follows. A stainless steel fixed-bed reactor with a length of 12 cm and an internal diameter of 0.72 cm was filled with *ca.* 600 g of crushed catalyst diluted in inert spheres. The catalyst used was the same as in the sorption/reactive regeneration experiments, 0.5 % ruthenium on alumina from Sigma-Aldrich. The filled reactor was placed inside a tubular oven in an experimental set-up described elsewhere.[1] For this experiment, a controlled evaporator mixer (model W-102A from Bronkhorst-High Tech) was used to evaporate H_2O and mix it into the inlet stream when desired. The composition of the dried outlet stream was measured in the equipment used in the sorption/reactive regeneration tests. Before the experiment was initiated, the catalyst was reduced in situ at 350 °C for 4 hours, with a stream composed of 30 $mL_N{\cdot}min^{-1}$ of H_2 and 70 $mL_N{\cdot}min^{-1}$ of N_2.

During the test, firstly a stream with 20 $mL_N{\cdot}min^{-1}$ of H_2O, 60 $mL_N{\cdot}min^{-1}$ of CH_4 and 20 $mL_N{\cdot}min^{-1}$ of N_2 was fed to the reactor. Then, i.e., after reaching steady-state, the H_2O flow was stopped (the inlet stream was composed solely of CH_4 and N_2). The outlet fraction of each component in the outlet stream (dry basis) is presented in Figure S.1, accompanied by the inlet conditions of each step of the experiment (above the graph).

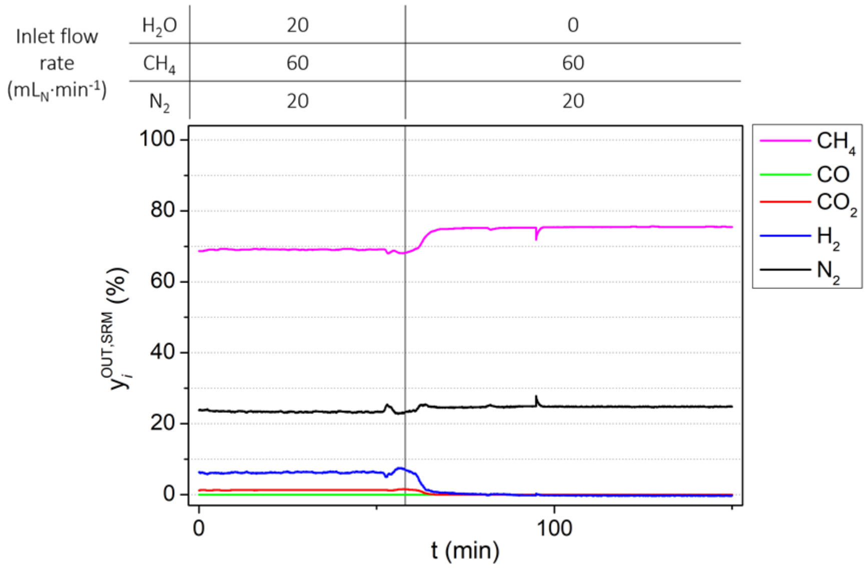

Figure S.1 – Outlet fraction of each component in the outlet stream (measured on dry basis) and inlet conditions of the CH_4 steam reforming experiment.

Figure S.1 shows that initially, as methane was fed simultaneously with water, the dried outlet stream contained CH_4 (*ca.* 70 %) that was not converted, but also H_2 and CO_2. As the water was stopped being fed, the H_2 and CO_2 outlet content decreased, eventually being null (or below the detection limit of the analyzer), and the outlet faction of CH_4 rose to *ca.* 75 % (the composition of the inlet stream). The outlet content of CO was null during the entire experiment.

Thus, it is possible to infer that the production of H_2 and CO_2 was related to the presence of H_2O, since both species were only detected when H_2O was being fed. It might be concluded that the steam reforming of methane can occur at 350 °C, being followed by water gas shift, producing H_2 and CO_2, even if to a very low extent. The consumption of CH_4 reached its highest value during the initial 50 minutes of this experiment. Still, the converted fraction was only *ca.* 1.8 % of the amount of CH_4 fed. In Figure S.1 it is possible to observe that during this initial step, the outlet content of H_2 was *ca.* 6 %. According to the overall stoichiometry of the steam reforming of CH_4 followed by water gas shift (Eq. (16) plus Eq. (17)), the consumption of one mole of CH_4 produces four moles of H_2, meaning that even if the reaction occurs to a small extent, the amount of H_2 formed can severely influence the purity of the outlet stream.

Parametric Study – Studies a to e

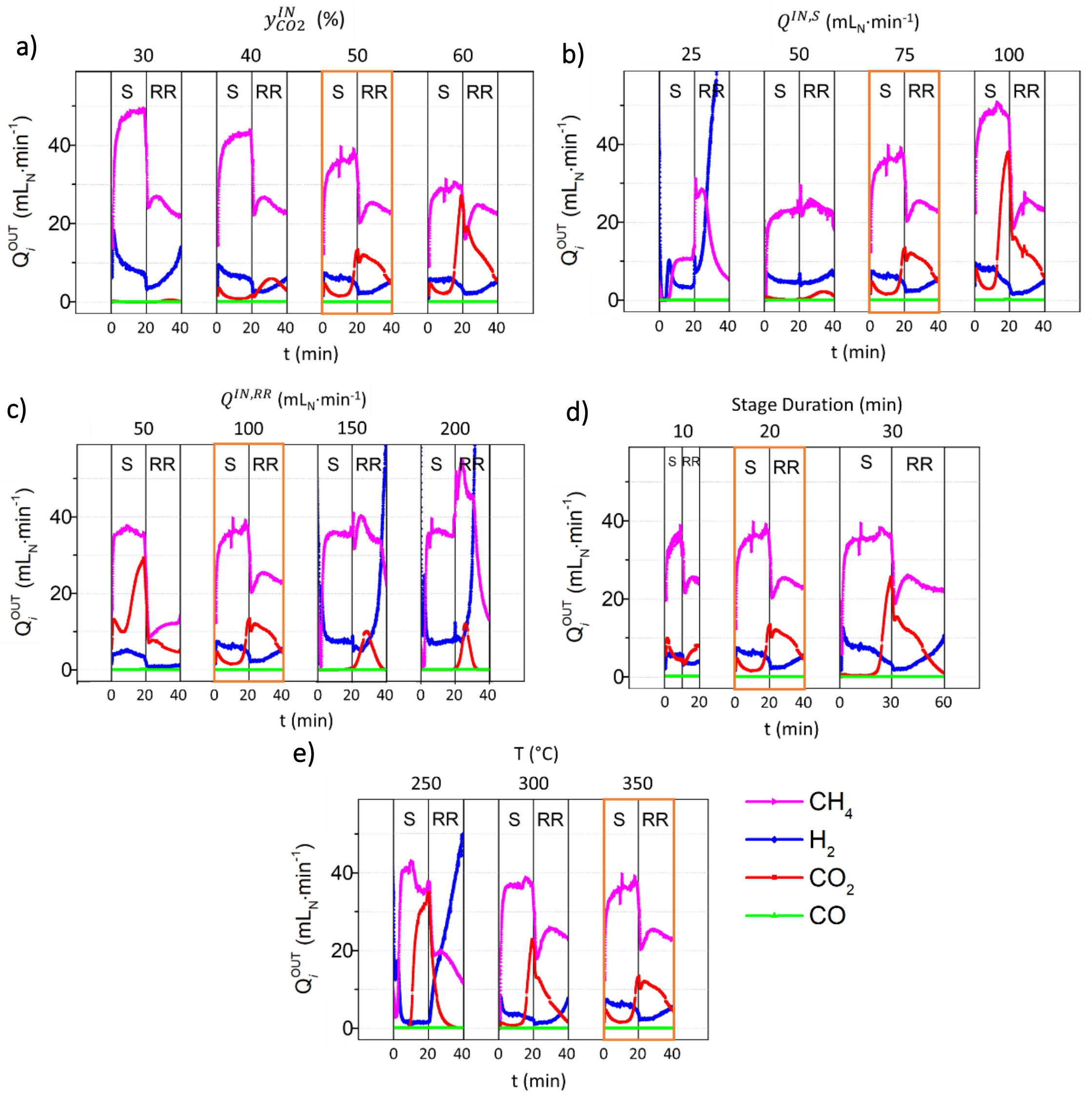

Figure S.2 – Effect of a) the inlet CO_2 content (Study a), b) the inlet flow rate during the sorption stage (Study b), c) the inlet flow rate during the reactive regeneration stage (Study c), d) the stage duration (Study d) and e) the temperature (Study e) on partial flow rate of the outlet streams at cyclic steady-state. The *S* and *RR* above each stage indicate whether it is a sorption (S) or reactive regeneration (RR) stage. Experimental conditions are given in Table 1. Orange frames mark the Reference experiment.

Table S.1- Effect of the inlet CO_2 content on process indicators (Study a).

y_{CO2}^{IN} (%)	q_{CO_2} (mol$_{CO2}$·kg$_{ads}$$^{-1}$)	X_{CO_2} (%)	$Prod_{CH_4}$ (mol$_{CH4}$·kg$_{cat}$$^{-1}$·h^{-1})	$\frac{n_{H_2}^{IN}}{n_{CH_4}^{prod}}$ (mol$_{H2}$·mol$_{CH4}$$^{-1}$)	$y_{CH_4}^{OUT}$ (%)
30	0.160	98.7	0.95	5.62	81.2
40	0.204	85.2	1.07	4.96	80.0
50	0.244	72.8	1.11	4.81	72.7
60	0.270	66.6	1.10	4.86	64.3

Table S.2 - Effect of the inlet flow rate during sorption stage on process indicators (Study b).

$Q^{IN,S}$ (mL$_N$·min^{-1})	q_{CO_2} (mol$_{CO2}$·kg$_{ads}$$^{-1}$)	X_{CO_2} (%)	$Prod_{CH_4}$ (mol$_{CH4}$·kg$_{cat}$$^{-1}$·h^{-1})	$\frac{n_{H_2}^{IN}}{n_{CH_4}^{prod}}$ (mol$_{H2}$·mol$_{CH4}$$^{-1}$)	$y_{CH_4}^{OUT}$ (%)
25	0.089	100.0	0.60	8.84	35.3
50	0.175	94.5	1.07	4.94	78.7
75	0.244	72.8	1.11	4.81	72.7
100	0.266	67.7	1.07	4.99	66.8

Table S.3 - Effect of the inlet flow rate during reactive regeneration stage on process indicators (Study c).

$Q^{IN,RR}$ (mL$_N$·min^{-1})	q_{CO_2} (mol$_{CO2}$·kg$_{ads}$$^{-1}$)	X_{CO_2} (%)	$Prod_{CH_4}$ (mol$_{CH4}$·kg$_{cat}$$^{-1}$·h^{-1})	$\frac{n_{H_2}^{IN}}{n_{CH_4}^{prod}}$ (mol$_{H2}$·mol$_{CH4}$$^{-1}$)	$y_{CH_4}^{OUT}$ (%)
50	0.144	66.5	0.49	5.47	61.3
100	0.244	72.8	1.11	4.81	72.7
150	0.267	86.8	1.55	5.12	70.3
200	0.267	91.4	1.63	6.54	49.9

Table S.4 - Effect of the stage duration on process indicators (Study d).

Stage duration (min)	q_{CO_2} (mol$_{CO2}$·kg$_{ads}$$^{-1}$)	X_{CO_2} (%)	$Prod_{CH_4}$ (mol$_{CH4}$·kg$_{cat}$$^{-1}$·h^{-1})	$\frac{n_{H_2}^{IN}}{n_{CH_4}^{prod}}$ (mol$_{H2}$·mol$_{CH4}$$^{-1}$)	$y_{CH_4}^{OUT}$ (%)
10	0.112	80.4	1.10	4.81	73.5
20	0.244	72.8	1.11	4.81	72.7
30	0.348	72.9	1.07	4.96	70.0

Table S.5 - Effect of the temperature on process indicators (Study e).

Temperature (°C)	q_{CO_2} (mol$_{CO2}$·kg$_{ads}$$^{-1}$)	X_{CO_2} (%)	$Prod_{CH_4}$ (mol$_{CH4}$·kg$_{cat}$$^{-1}$·h^{-1})	$\frac{n_{H_2}^{IN}}{n_{CH_4}^{prod}}$ (mol$_{H2}$·mol$_{CH4}$$^{-1}$)	$y_{CH_4}^{OUT}$ (%)
250	0.173	75.0	0.71	7.48	49.4
300	0.235	76.5	1.12	4.75	75.6
350	0.244	72.8	1.11	4.81	72.7

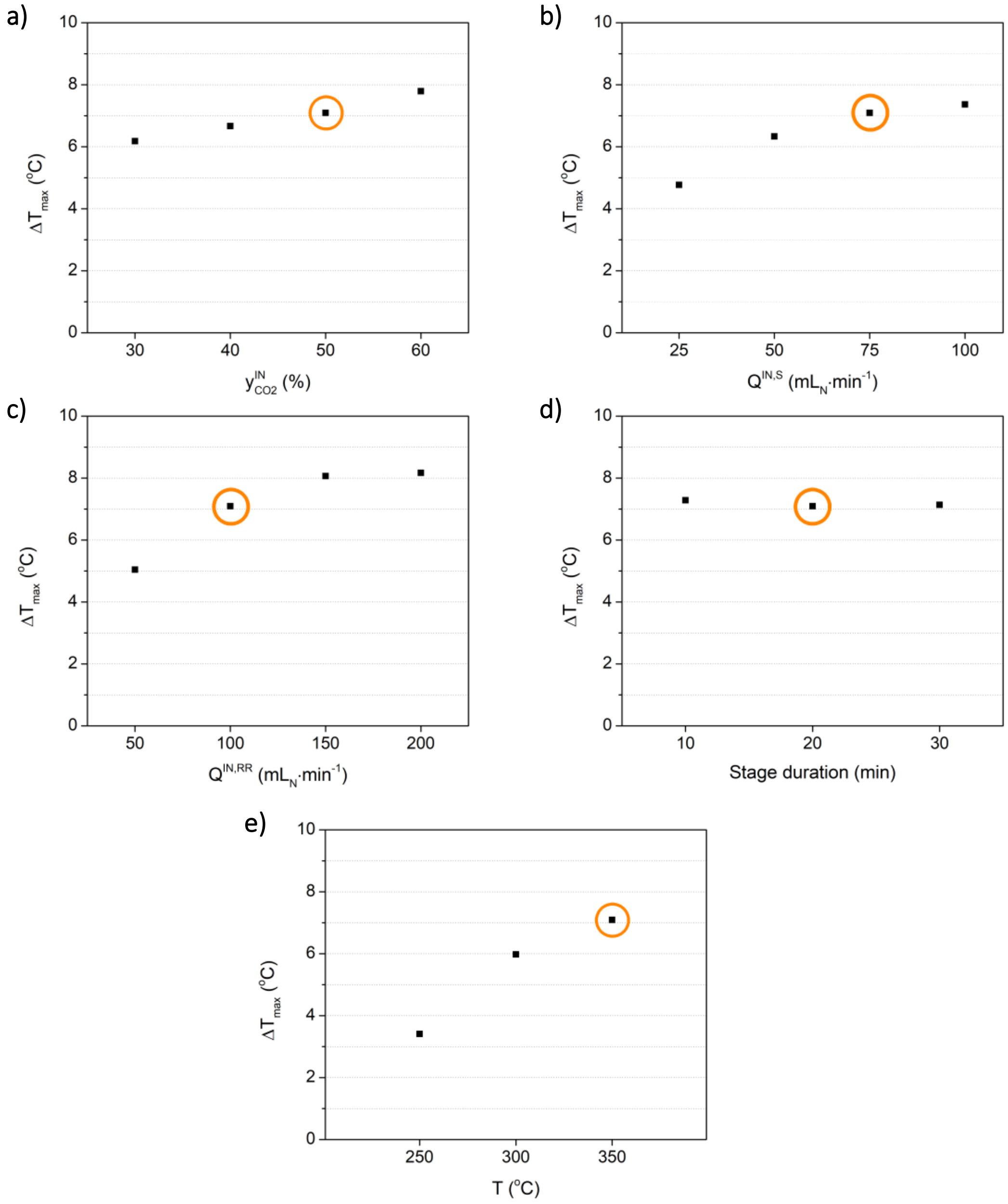

Figure S.3 – Effect of a) the inlet CO_2 content (Study a), b) the inlet flow rate during the sorption stage (Study b), c) the inlet flow rate during the reactive regeneration stage (Study c), d) the stage duration (Study d) and e) the temperature (Study e) on maximum bed temperature variation (at steady-state). Experimental conditions are given in Table 1. Orange circles mark the Reference experiment.

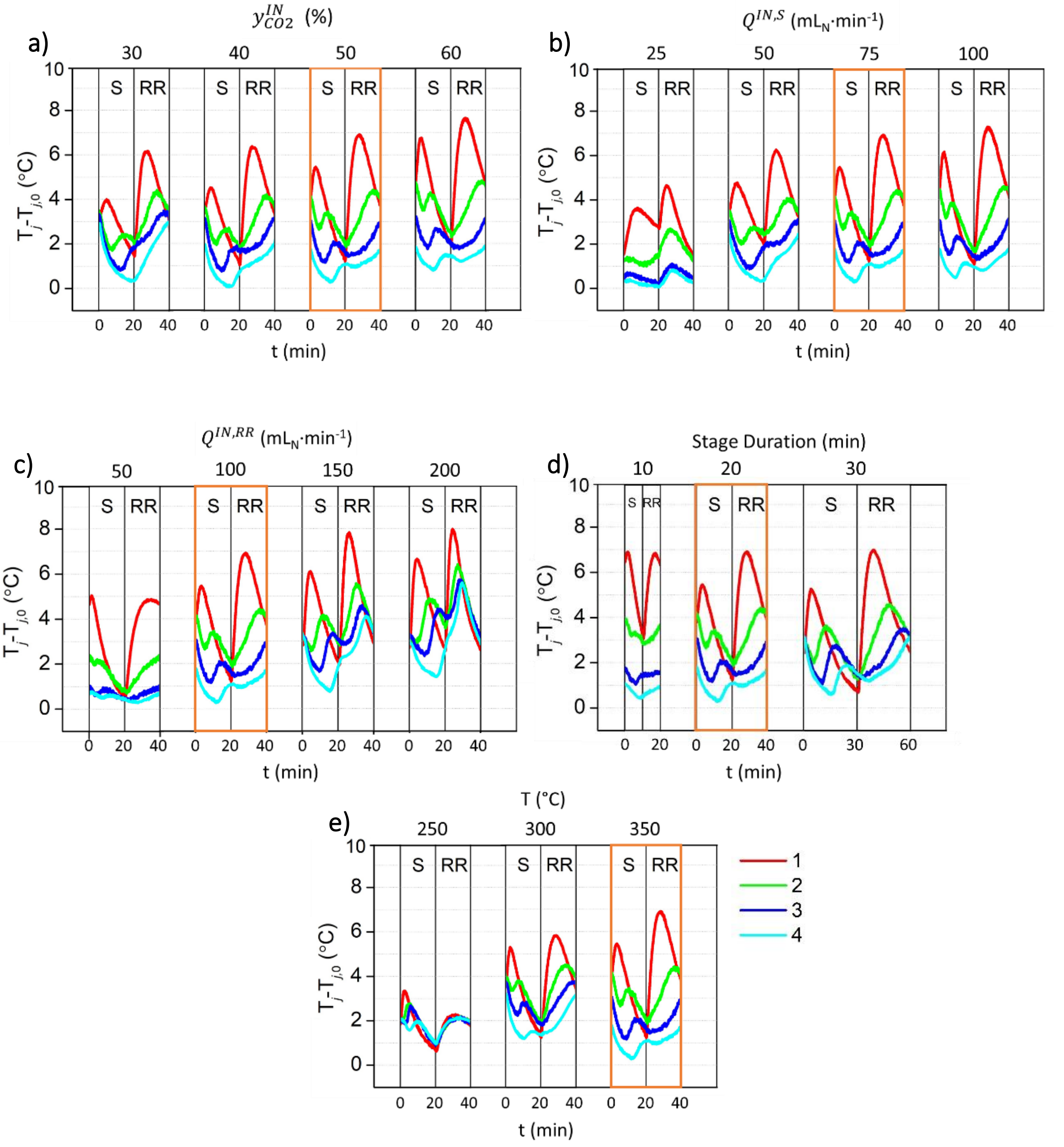

Figure S.4 - Effect of a) the inlet CO_2 content (Study a), b) the inlet flow rate during the sorption stage (Study b), c) the inlet flow rate during the reactive regeneration stage (Study c), d) the stage duration (Study d) and e) the temperature (Study e) on normalized bed temperature (T_1 read on the thermocouple closest to the reactor inlet and T_4 closest to the outlet) at cyclic steady-state. The S and RR above each stage indicate whether it is a sorption (S) or reactive regeneration (RR) stage. Experimental conditions are given in Table 1. Orange frames mark the Reference experiment.

Parametric Study – Purge Stage – Study f

Study f aimed to assess the effect of adding a purge stage to the sorption/reactive regeneration cycles. The first experiment with purge stage (*P*) was performed in the same operating conditions as the Reference experiment (*cf.* Table 1), except for the added purge stage. The results obtained are presented in Table S.6 and Figure S.5 alongside the equivalent experiment without purge.

Table S.6 shows that the addition of a purging step after reactive regeneration (*RR*) benefited the amount of captured CO_2 since the flushing of the sorbent with N_2 allowed for the remaining CO_2 to be desorbed (*cf.* red bar in the purge stage (*P*) in Figure S.5 a)), ensuring a better regeneration of the sorbent. Still, although the amount of CO_2 sorbed was greater, due to the limited amount of H_2 available, the CO_2 conversion was lower in the experiment with purge, resulting in very similar outlet streams during the reactive regeneration stages (*cf.* *RR* stages Figure S.5 a) and b)).

Table S.6 - CO_2 sorption capacity, CO_2 conversion, CH_4 productivity, moles of H_2 fed per mole of CH_4 produced and average methane outlet fraction during S/RR, obtained during the Reference experiment with and without purge stage.

Experiment	$q_{\mathrm{CO_2}}$ ($\mathrm{mol_{CO2}\cdot kg_{ads}^{-1}}$)	$X_{\mathrm{CO_2}}$ (%)	$Prod_{\mathrm{CH_4}}$ ($\mathrm{mol_{CH4}\cdot kg_{cat}^{-1}\cdot h^{-1}}$)	$\frac{n_{H_2}^{IN}}{n_{\mathrm{CH_4}}^{prod}}$ ($\mathrm{mol_{H2}\cdot mol_{CH4}^{-1}}$)	$y_{\mathrm{CH_4}}^{OUT}$ (%)
NO PURGE	0.244	72.8	1.11	4.81	72.7
PURGE	0.260	67.7[a]	0.64[b]	5.55[c]/5.11[d]	68.7[e]

[a] to calculate the $X_{\mathrm{CO_2}}$ in the experiment with purge, a parcel accounting for the CO_2 that exited the sorptive reactor during the purge step was added to the numerator of Eq.(3).
[b] the $Prod_{\mathrm{CH_4}}$ of the experiment with purge calculated by Eq. (4), but considering also the duration of the purge stage (added to the denominator), *i.e.*, the CH_4 that exited the sorptive reactor during the purge step was not considered.
[c] calculated according to Eq. (6).
[d] value if the amount of CH_4 that exited the sorptive reactor during the purge stage was considered.
[e] calculated according to Eq. (9), but with N_2 as a possible component. The composition of the outlet stream of the purge stage was not considered.

For the calculation of productivity in Table S.6, the CH_4 that exited the sorptive reactors in the purge stage was not considered (since it is highly diluted in N_2). However, the duration of the purge stage was taken into account, as it would take the additional 20 minutes of purge to produce the CH_4. The addition of a purge stage negatively influenced such indicator ($Prod_{\mathrm{CH_4}}$), which decreased from 1.11 (without purge) to 0.64 $mol_{CH4} \cdot kg_{cat}^{-1} \cdot h^{-1}$ (with purge). From the analysis of Table S.6, it is possible to conclude that the addition of a purge stage also compromised the amount of hydrogen required ($\frac{n_{H_2}^{IN}}{n_{\mathrm{CH_4}}^{prod}}$) and methane purity ($y_{\mathrm{CH_4}}^{OUT}$), although the variation was not very significant. The methane purity was calculated considering only the *S* and *RR* stages, even in the experiment with the purge.

Although the methane purity obtained with and without purge was similar (68.7 % and 72.7 %, respectively), Figure S.5 a) demonstrates that there were some differences in the content of the outlet streams of both tests, particularly in the sorption stage. The outlet stream of the sorption stage was richer in CH_4 without purge, with the second and third major components being H_2 and CO_2, respectively. However, in the experiment with purge, the outlet stream was composed mostly of CH_4 but also N_2, H_2, and CO_2, in this order.

The purge stage allowed the removal of CO_2 (*c.f.* red line in purge stage (*P*) of Figure S.5 b)) and H_2O (not measured) that remained in the sorptive reactors from the reactive regeneration. During sorption stage, the presence of less H_2O inside the packed bed inhibited the occurrence of steam reforming of methane followed by water gas shift reaction, which resulted in the decrease of the outlet flow rate of H_2 and CO_2 (*c.f.* sorption stages in Figure S.5 b)). The reduction of CO_2 outlet flow rate was also caused by the better regeneration of the sorbent (due to CO_2 desorption during purge). However, despite the expected beneficial influence of the purge on the CH_4 purity during the sorption stage (due to the mitigation of SRM and enhanced regeneration of sorbent), the N_2 (fed during purge) that exited the reactor during the sorption stage decreased the CH_4 purity, ultimately resulting in lower $y_{\mathrm{CH_4}}^{OUT}$ (in comparison with the experiment without purge). In Figure S.5 a) and b) it is possible to confirm the negative influence of the N_2 on the outlet stream of the experiment with the purge.

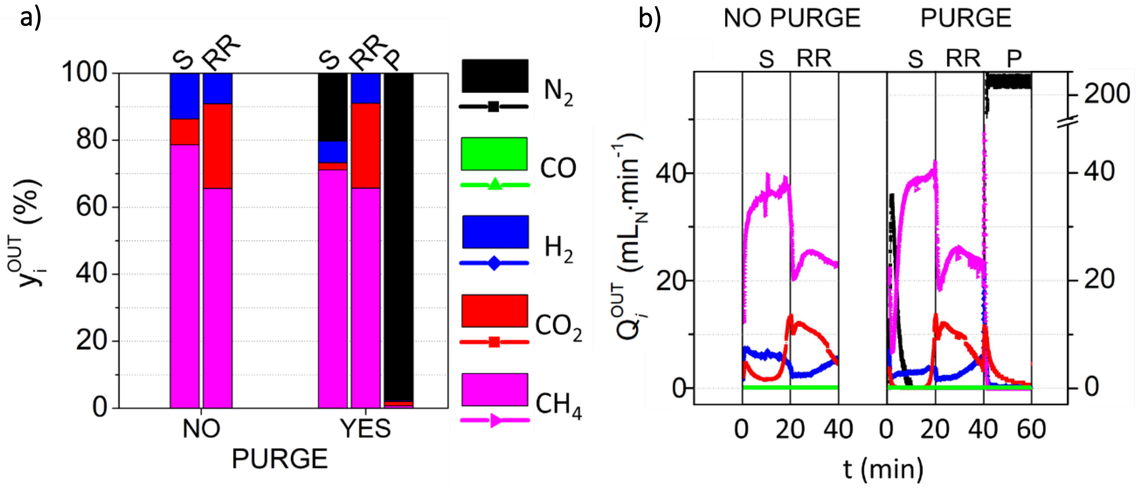

Figure S.5 – a) average outlet fraction and b) partial outlet flow rate of all components (CH_4, CO_2, H_2, CO, and N_2) during the sorption stage (S), reactive regeneration stage (RR) and purge stage (P) of the Reference experiment (purge - no), and the Reference experiment with purge (purge – yes). Experimental conditions are given in Table 1.

The second test with a purge stage was similar to the experiment already discussed in Study c performed with a regenerative flow rate of 50 $mL_N \cdot min^{-1}$ (*cf.* Table 1), with the addition of the 20-minute N_2 flushing at the end of the reactive regeneration stage. This experiment was chosen because it was the one with the highest CO_2 and CO outlet content during the sorption stage. The results obtained in the experiment with purge are presented in Table S.7 and Figure S.6 alongside the results of the experiment without pure (already presented during the discussion of Study c).

In Table S.*7* it is possible to observe that the inclusion of a purge step had an effect, on most process indicators, similar to the one observed in the Reference experiment. The amount of captured CO_2 was increased (due to better sorbent regeneration), and both CO_2 conversion and methane productivity decreased, while CH_4 purity was almost the same. Regarding the amount of consumed hydrogen ($\frac{n_{H_2}^{IN}}{n_{\mathrm{CH_4}}^{prod}}$), if the methane that exited during the purge stage is taken into consideration, the value obtained is lower in the experiment with purge (4.88 $mol_{H2}\cdot mol_{CH4}^{-1}$, *cf.* Table S.7) than without purge (5.47 $mol_{H2}\cdot mol_{CH4}^{-1}$). However, if this amount of CH_4 (which cannot be used due to high dilution in N_2) is not taken into account, the indicator obtained is much higher (8.77 $mol_{H2}\cdot mol_{CH4}^{-1}$). This is because, as the productivity in these experiments is low, the methane that remains in the packed bed from the reactive regeneration to the purge becomes significant (*cf.* pink bar in the purge stage (*P*) in Figure S.6 a)).

Regarding CO formation, the addition of the purge stage did not impede its production which was observed at the end of the sorption stage. Nonetheless, during sorption stage, the outlet flowrate of H_2 and CO_2 was lower in the experiment with purge than in the one without (*cf.* Figure S.6 b)). This means that, even though the steam reforming of methane and following water gas shift reaction were partly mitigated (due to H_2O removal during purge), there was still some CO that was formed by SRM but that was not further converted. In the experiment with N_2 flushing, the average CO content in the outlet stream during S/RR was 502 ppm (while in the experiment without purge it was 392 ppm).

Table S.7 - CO_2 sorption capacity, CO_2 conversion, CH_4 productivity, moles of H_2 fed per mole of CH_4 produced and average methane outlet fraction during S/RR, obtained during the experiment with $Q^{IN,RR}$ of 50 $mL_N\cdot min^{-1}$ with and without purge stage.

Experiment	$q_{\mathrm{CO_2}}$ ($mol_{CO2}\cdot kg_{ads}^{-1}$)	$X_{\mathrm{CO_2}}$ (%)	$Prod_{\mathrm{CH_4}}$ ($mol_{CH4}\cdot kg_{cat}^{-1}\cdot h^{-1}$)	$\frac{n_{H_2}^{IN}}{n_{\mathrm{CH_4}}^{prod}}$ ($mol_{H2}\cdot mol_{CH4}^{-1}$)	$y_{\mathrm{CH_4}}^{OUT}$ (%)
NO PURGE	0.144	66.5	0.49	5.47	61.3
PURGE	0.214	46.2[a]	0.21[b]	8.77[c]/4.88[d]	61.6[e]

[a] to calculate the $X_{\mathrm{CO_2}}$ in the experiment with purge, a parcel accounting for the CO_2 that exited the sorptive reactor during the purge step was added to the numerator of Eq. (3).
[b] the $Prod_{\mathrm{CH_4}}$ of the experiment with purge calculated by Eq. (4), but considering also the duration of the purge stage (added to the denominator), *i.e.*, the CH_4 that exited the sorptive reactor during the purge step was not considered (as it would not be usable due to its severe dilution in N_2).
[c] calculated according to Eq.(6).
[d] value if the amount of CH_4 that exited the sorptive reactor during the purge stage was considered.
[e] calculated according to Eq.(9), but with N_2 as a possible component. The composition of the outlet stream of the purge stage was not considered.

In Figure S.6 it is possible to observe that, regarding CH_4 purity, the addition of the purge stage after the reactive regeneration had the same effect as in the Reference experiment with/without purge (H_2 and CO_2 outlet content in the sorption stage was reduced, but there was N_2 dilution).

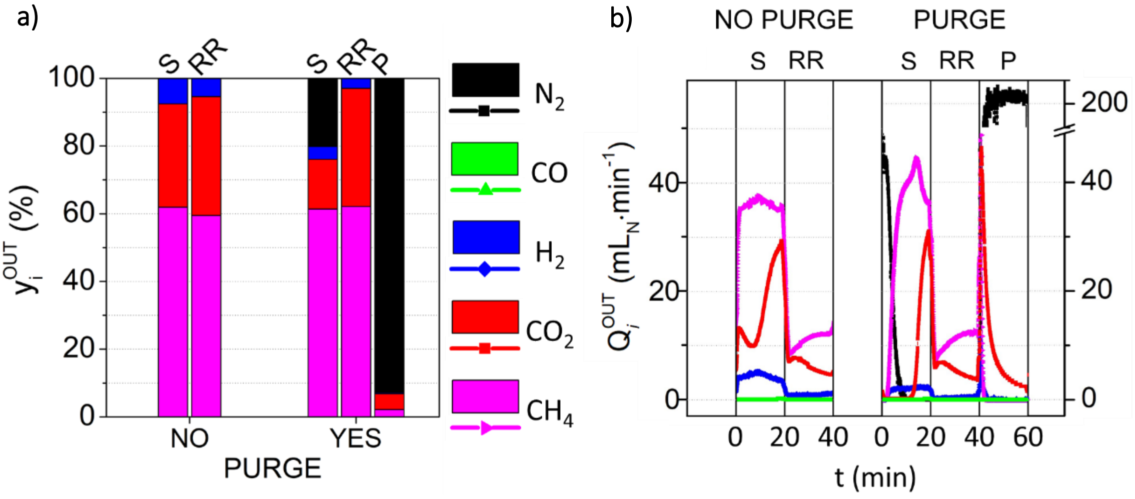

Figure S.6 – a) average outlet fraction and b) partial outlet flow rate of all components (CH_4, CO_2, H_2, CO, and N_2) during the sorption stage (S), reactive regeneration stage (RR) and purge stage (P) of the experiment with $Q^{IN,RR}$ of 50 $mL_N \cdot min^{-1}$ without purge (purge - no) and with purge (purge – yes). Experimental conditions are given in Table 1.

In conclusion, the addition of an N_2 flushing step did not have a very positive impact on the process indicators. Additionally, it is important to consider that the use of inert gas, N_2 in this case, would also increase the operating cost of the process. Nevertheless, the experiments performed with purge allow understanding that a "cleaning" stage after reactive regeneration could be beneficial for both the mitigation of CH_4 consumption by SRM and for sorbent regeneration. For instance, if the whole process was carried out at higher pressure, a purge stage in which the total pressure was reduced, leading to H_2O and CO_2 desorption, would present the advantages of the N_2 flushing, without compromising the CH_4 purity during the sorption stage (as there would be no inert species involved). Furthermore, the performance of a faster purge stage executed by variation of total pressure could also reduce the negative impact that the elongation of the cycle has on productivity.

Comparison of direct hydrogenation with the cyclic unit

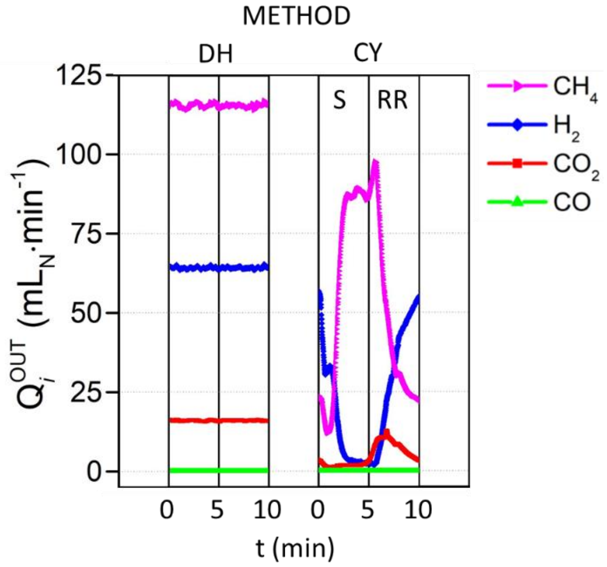

Figure S.7 - Partial outlet flow rate of each component (at steady-state) during test number 4 of Table 3, obtained with the direct hydrogenation (DH) and cyclic method (CY). The *S* and *RR* above each stage indicate whether it is a sorption (S) or reactive regeneration (RR) stage.

Nomenclature

Symbols (unit)

ΔT_{max}	Maximum bed temperature variation at steady-state (°C)
$\frac{n_{H_2}^{IN}}{n_{CH_4}^{prod}}$	Moles of hydrogen fed per mole of methane produced ($mol_{H2}\cdot mol_{CH4}^{-1}$)
$Prod_{CH_4}$	Methane productivity ($mol_{CH4}\cdot kg_{cat}^{-1}\cdot h^{-1}$)
q_{CO_2}	Carbon dioxide sorption capacity ($mol_{CO2}\cdot kg_{ads}^{-1}$)
$Q^{IN,RR}$	Inlet flow rate during reactive regeneration stage ($mL_N\cdot min^{-1}$)
$Q^{IN,S}$	Inlet flow rate during sorption stage ($mL_N\cdot min^{-1}$)
Q_i^{OUT}	Outlet flow rate of component *i* ($mL_N\cdot min^{-1}$)
T	Oven temperature (°C)
T_j	Temperature read on thermocouple *j* (°C)
$T_{j,0}$	Initial temperature read on thermocouple *j* (°C)
t	Time (min)
X_{CO_2}	Carbon dioxide conversion (%)
$y_{CO_2}^{IN}$	Inlet CO_2 content (%)
y_i^{OUT}	Average outlet fraction of component *i* during a full cycle (%)
$y_i^{OUT,SMR}$	Outlet fraction of component *i* during the steam reforming of methane experiment (%)

Subscripts and superscripts

0	Initial
i	Components CH_4, CO_2, H_2, CO and N_2
IN	Sorptive reactor inlet
j	Thermocouples 1,2,3 and 4
max	Maximum value
OUT	Sorptive reactor outlet
RR	Reactive regeneration stage
S	Sorption stage
SRM	Steam reforming of methane experiment

References

(1) Martins, J. A.; Faria, A. C.; Soria, M. A.; Miguel, C. V.; Rodrigues, A. E.; Madeira, L. M. CO2 Methanation over Hydrotalcite-Derived Nickel/Ruthenium and Supported Ruthenium Catalysts. *Catalysts* **2019**, *9*, 1008.
